# Experimental characterization and machine learning modeling of leakage-induced soil fluidization in water distribution systems

**DOI:** 10.1371/journal.pone.0331097

**Published:** 2025-09-23

**Authors:** Masoud Ghodsian, Shima Mohammadbeigi

**Affiliations:** Faculty of Civil and Environmental Engineering, Tarbiat Modares University, Tehran, Iran; University of Oklahoma, UNITED STATES OF AMERICA

## Abstract

Leakage in water distribution systems poses a global challenge, not only due to resource loss but also through soil erosion and sinkhole formation, which risk infrastructure collapse. This study investigates the mechanisms of soil fluidization, a process in which pressurized pipeline leakage generates turbulent water-soil mixtures, forming expanding fluidized zones. Experimental tests using a custom leakage simulation apparatus, combined with dimensional analysis, were conducted to identify key factors influencing fluidization dynamics. Empirical equations were developed to predict fluidized zone height and area (R² = 0.753–0.915) for both upward and downward leakages. These models were validated against 150 experimental datasets from current and prior studies, covering a wide range of leakage rates and soil types. Three ensemble machine learning models—Random Forest, XGBoost, and a Stacking model integrating support vector regression, multilayer perceptron, and linear regression—were employed to enhance predictive accuracy and stability. The results of evaluation metrics (R^2^, RMSE and correlation coefficient) showed that although XGBoost outperformed other models regarding accuracy (with R^2^ = 0.91 in test splits), this model exhibited the lowest stability in predicting dimensionless height and area of fluidized zone (with ΔR² = 0.07–0.08). The Random Forest model had the lowest accuracy (R^2^ = 0.907–0.912 in train phase) though the most capability in generalization through the minimum differences between train and test splits (with ΔR² = 0.02–0.04). Regarding Stacking model, both accuracy and stability maintained in balanced conditions with moderate performance. The conclusion from findings of all evaluation metrics were the same as deterministic coefficient. The equations derived from dimensional analysis, especially equations for downward leakage direction, also showed comparable performance with the most accurate ensemble model like XGBoost (with R² up to 0.915). Temporal analysis revealed the progression of fluidization through seven distinct stages over approximately 75 seconds. Fluidization initiated at a specific discharge rate, beginning with the formation of a 2 cm cavity and culminating in vortex bifurcation and dimensional stabilization. Critical pore pressure thresholds, observed around 20 seconds, induced suspended particle states and fountain flow, while vertical cavity growth predominated between 25 and 65 seconds. Sensitivity analysis highlighted the densimetric Froude number and soil uniformity as dominant factors, with their omission reducing model R² by up to 84.1% and 71.3%, respectively. In contrast, the particle size-to-leak area ratio exhibited marginal effect (<10% reduction). These findings provide critical insights into sinkhole risk mitigation, offering practical tools for infrastructure vulnerability assessment.

## 1. Introduction

Leakage in buried water distribution systems not only results in considerable water loss but also leads to subsoil erosion, which can trigger sinkhole formation and damage critical infrastructure. These failures pose significant safety and economic risks. In China, about 55% of urban sinkholes are attributed to pipeline leakage-induced erosion [1], with similar incidents reported globally [2], highlighting the importance of understanding soil-fluid interactions under leak conditions.

Internal erosion, especially in the form of fluidization, is a key mechanism by which pressurized leaks mobilize soil particles and form expanding cavities [3–5]. These fluidized zones can progress rapidly and breach the surface, leading to catastrophic collapse. Several numerical and laboratory studies have characterized fluidization in stages—from non-fluidized buildup to cavity breakthrough—emphasizing the complexity of the process [6–9]. Other works have observed both cylindrical and conical cavity geometries with distinct particle motion patterns, revealing inconsistencies that demand further investigation [10–12].

Although some progress has been made in modeling fluidization, most studies have generalized the process or focused on transient flows, while steady flow conditions—common in real pipeline failures—have received limited attention. Moreover, cavity growth is influenced by factors such as leakage geometry, soil characteristics, hydraulic gradient, and burial depth [13–17]. Existing empirical models provide partial estimates of cavity dimensions [18,19], but a comprehensive predictive framework is still lacking. The influence of orifice size also remains debated: some findings suggest negligible impact on critical thresholds [8], while others show significant effects depending on flow regime and soil depth [5,11,20].

In recent years, machine learning (ML) has emerged as a powerful tool for modeling complex, nonlinear processes in hydraulic engineering. ML algorithms, particularly ensemble methods like Random Forest, XGBoost, and Stacking, have demonstrated superior predictive capabilities over traditional models in applications such as flow velocity prediction [21], scour estimation [22–29], spillway hydraulics [30–32], and wave-structure interactions [33,34]. These models reduce overfitting, handle high-dimensional data, and generalize well to real-world variability [35–37]. However, ML techniques have not yet been applied to predict soil fluidization in leak scenarios.

This study addresses these gaps by: (1) experimentally investigating fluidization under steady leak conditions, (2) developing empirical equations for fluidized zone dimensions, and (3) applying ensemble ML models to improve prediction accuracy and support sinkhole risk assessment in buried pipeline systems. By combining experimental data analysis with empirical modeling, this study establishes quantitative relationships between key parameters governing soil-fluid interactions. The resulting framework provides practical tools for identifying erosion-prone zones and mitigating sinkhole risks in urban infrastructure systems.

## 2. Materials and methods

### 2.1. Experimental setup

**Fig 1:**
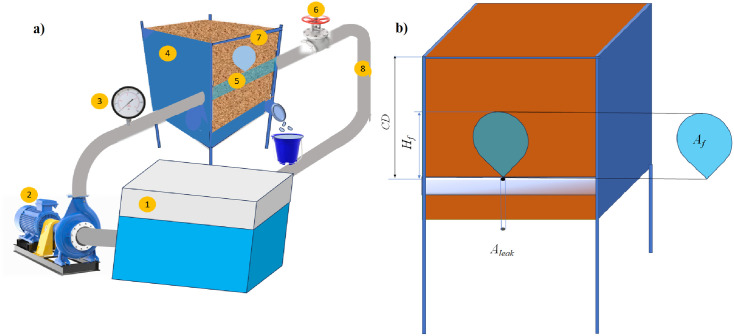
(a) Schematic of the leakage simulation apparatus: 1 – Water supply tank, 2 – Water pump, 3 – Pressure gauge, 4 – Soil containment box with a leaking pipe, 5 – Primary pipeline with an upward-directed leakage, 6 – Pressure control valve, 7 – Leakage water outlet, 8 – Pipes and fittings. (b) Measured parameters in the experiments.

A custom-designed leakage simulation apparatus was developed to replicate soil fluidization induced by pressurized leakage in buried water pipelines. The primary objective was to investigate the geometric characteristics of the fluidized region under controlled laboratory conditions (Fig 1). The apparatus comprised several key components: (1) a water supply system, including an 800-liter storage tank, a centrifugal pump capable of delivering up to 2.67 × 10 ⁻ ³ m³/s discharge, and an oil-filled pressure gauge; (2) a soil containment box, with internal dimensions of 25 × 20 × 30 cm (length × width × height), fitted with an outlet for discharge collection; (3) a pipeline and leakage simulation system, consisting of a 4 cm diameter pipe embedded horizontally within the soil and equipped with a 3 mm diameter circular orifice at its crown to simulate leakage; (4) a flow control and measurement system, including a control valve and associated fittings for regulating the flow rate; and (5) a visualization and measurement system, featuring a 10 cm thick transparent glass panel on the front face of the soil box, allowing clear observation and manual tracing of the fluidized region’s height (*H*_*f*_) and area (*A*_*f*_). Visual data were extracted from high-frame-rate video recordings (see S1 Video in the Supporting information), which captured the entire fluidization process from initiation to full development. Specific frames corresponding to threshold moments were selected for analysis. Due to the well-defined progression of the process in distinct stages, reducing time intervals further did not yield additional transitions. Leakage discharge was determined using a volumetric method.

**Fig 2:**
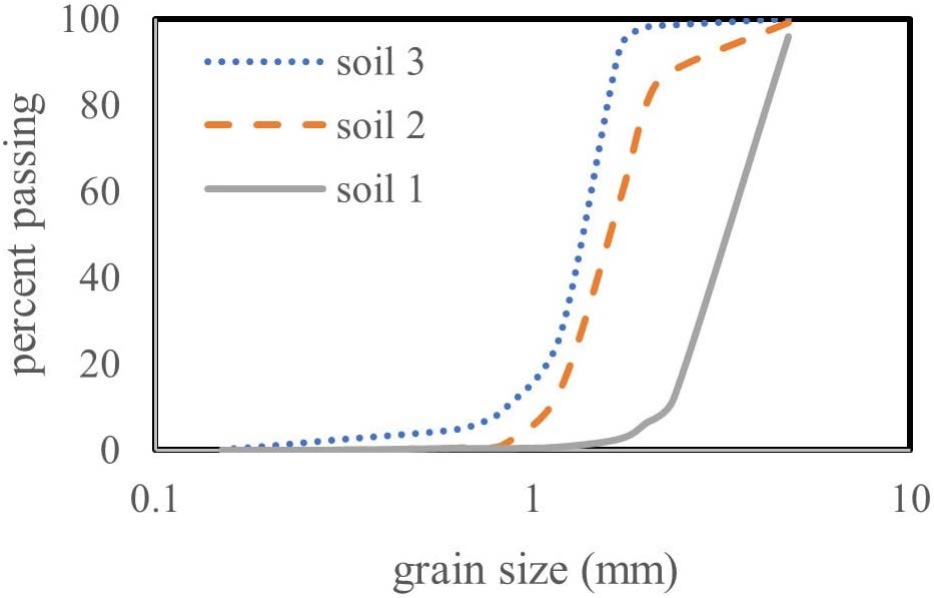
Grain size distribution curves of the soils used in the experiments.

Three types of granular soil, differing in median particle diameter (*d*_50_) and uniformity coefficient (*Cu* = *d*_60_/*d*_10_), were used to simulate the surrounding soil medium. The grain size distribution curves and characteristics of these soils are presented in Fig 2 and Table 1, respectively. The relative density (*G*) of the soil remained constant across all experiments.

**Table 1:** Characteristics of the soils used in the experiments.

Soil	*d*_50_	*d*_60_	*d*_10_	*Cu*	*G*
1	3.3	3.55	2.29	1.55	**2.7**
2	1.6	1.70	1.11	1.53	**2.7**
3	1.3	1.43	0.86	1.66	**2.7**

### 2.2. Experimental procedure

The experimental procedure began by fully opening the pressure control valve and activating the pump. The valve was then gradually adjusted until the desired pressure was achieved and stabilized. The system was left undisturbed to allow natural development of the fluidized zone. The entire process was continuously recorded using a high-resolution video camera to document fluidization dynamics. Leakage discharge for each test was determined by collecting the outflow over a fixed time period, weighing the water with a precision scale, and converting the mass to volume using water density. Discharge measurements were repeated at multiple intervals to ensure accuracy, with average values reported. Upon achieving full fluidization, the shape of the resulting fluidized zone was traced directly onto a transparent plastic sheet affixed to the front glass panel of the apparatus. After documenting the fluidized boundary, the pump was switched off, and the experimental sequence was repeated for the remaining test conditions.

The independent variables investigated in this study included leak discharge (*Q*_*leak*_), which was proportional to applied pressure levels of 1.5, 2.5, and 3.5 bars; soil cover depth (*CD*), tested at 380 mm and 400 mm; and soil type, comprising three granular soils with varying grain size distributions. These variables were selected to assess their influence on the onset and geometry of the fluidized region under controlled leakage scenarios.

### 2.3. Dataset compilation and preprocessing

In addition to the experimental data collected in this study, datasets from previous studies were incorporated to provide a comprehensive analysis. These datasets included:

1. **Upward-directed leakage**: A dataset of 81 data points is extracted from previous studies [38 and 39]. One, investigated the effects of soil cover depth, pressure, and soil type on fluidization [38]. Another study focused on leakage geometry and soil properties [39]. Both studies utilized polyethylene and uPVC pipes with leakage diameters of 40 mm.
2. **Downward-directed leakage**: A dataset of 69 data points is extracted from previous studies [40 and 41]. One, examined the influence of leakage geometry (crack vs. orifice) and soil type [40]. Another study analyzed the effects of soil non-uniformity and longitudinal crack size [41].

Input variables extracted from these studies included soil properties (*Cu*, *d*_50_, *CD*, *G*), leakage geometry (*A*_*leak*_), and flow characteristics (*V*_*inlet*_, calculated as *Q*_*leak*_*/A*_*leak*_) (“S1 Dataset”).

### 2.4. Dimensional analysis

Dimensional analysis was performed using the Buckingham П theorem to identify key dimensionless groups influencing fluidization, such as the densimetric Froude number, soil characteristics, cover depth and geometric ratios. The derivation of these groups and the full dimensional analysis are provided in “S2 Appendix”. Empirical relationships were derived through Python programming, with curve fitting performed via the SciPy library. General input data is in “S3 and S4 Dataset”; and source codes are in “S4 to S7 Code”. The accuracy of the resulting models was assessed using the coefficient of determination (R²), root mean square error (RMSE), and Taylor diagrams. Additionally, sensitivity analysis was carried out using the Leave-One-Out method to evaluate the influence of each input parameter on the model’s performance.

### 2.5. Machine learning modeling

**Fig 3:**
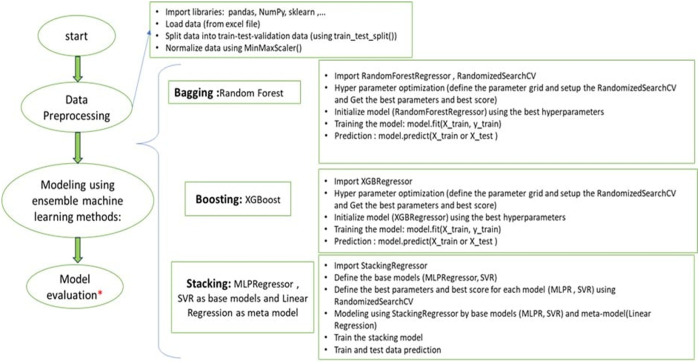
Workflow from data preprocessing to model development and evaluation.

Three ensemble machine learning models—Random Forest, XGBoost, and a Stacking model integrating support vector regression, multilayer perceptron, and linear regression—were developed to predict the relative height (*H*_*f*_*/d*_50_) and area (√*A*_*f*_/*d*_50_) of the fluidized zone for both upward and downward leakage directions. These models were selected based on their proven ability to capture complex, nonlinear relationships, reduce overfitting through ensemble strategies, and generalize well across varied datasets—characteristics particularly relevant to modeling the highly variable and nonlinear nature of fluid–soil interactions in civil engineering applications. Random Forest and XGBoost are widely recognized for their robustness, resistance to noise, and interpretability, especially in structured data domains. The inclusion of a Stacking model allows for combining the strengths of multiple base learners (SVR, MLP, and linear regression), potentially improving predictive accuracy through model diversity. Prior studies have demonstrated the efficacy of ensemble methods in geotechnical and hydraulic engineering contexts, further supporting their selection for this study [35–37]. Model development, training, and validation were implemented in Python using the scikit-learn and XGBoost libraries. To ensure predictive accuracy and generalizability, models were trained on experimental datasets derived through dimensional analysis (Eqs. 3 and 4). The workflow included comprehensive data preprocessing, hyperparameter optimization, and performance evaluation following established best practices (Fig 3). Model performance was assessed using three complementary metrices: coefficient of determination (R²), root mean square error (RMSE), and correlation coefficient (R). Together, these metrics provide a comprehensive evaluation of both accuracy and consistency across different leakage conditions. The input data are provided in “S4 Dataset” and the complete model source codes are available in “S8 to S13 Code”. This ensemble learning approach enabled robust prediction of fluidization behavior under varying soil and leakage conditions, demonstrating its effectiveness in capturing the underlying nonlinear dynamics of the fluid–soil system.

#### 2.5.1. Ensemble learning frameworks.

**Fig 4:**
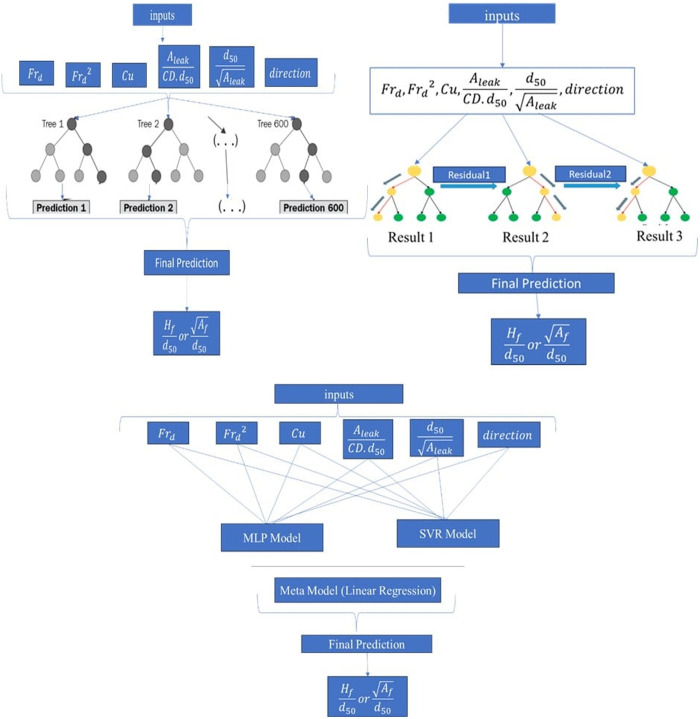
Algorithm of ensemble machine learning models used in: a) Bagging model (Random Forest), b) Boosting model (XGBoost), and c) Stacking model (MLP, SVR).

Random Forest (Bagging): A bootstrap aggregating (Bagging) algorithm was implemented using the *RandomForestRegressor* library. Multiple decision trees (*nestimators* = 100–500) were trained on bootstrapped subsets of the data, with predictions averaged to minimize variance and overfitting. Key hyperparameters included tree depth (*maxdepth* = 5–20) and node-splitting thresholds (**minsamples_split* *= 2–10 (Fig 4a) (see “S8 and S9 Code”).

XGBoost (Boosting): The Extreme Gradient Boosting (*XGBRegressor*) algorithm optimized prediction accuracy through sequential error correction. Hyperparameters such as learning rate (*η* = 0.01–0.3), tree depth (*maxdepth* = 3–10), and ensemble size (**nestimators* *= 100–500) were tuned to balance bias-variance trade-offs (Fig 4b) (see “S10 and S11 Code”).

Stacked Generalization: A two-stage meta-learner combined predictions from heterogeneous base models—Support Vector Regressor (SVR) (radial basis function kernel) and Multilayer Perceptron Regressor (*MLPRegressor*) (two hidden layers, ReLU activation)—using linear regression as the meta-model. This hybrid approach leveraged complementary strengths of parametric and non-parametric methods (Fig 4c) (see “S12 and S13 Code”).

#### 2.5.2. Data preprocessing.

The dataset (*n* = 150), comprising experimental results from current and prior studies, underwent three key preprocessing steps. First, stratified splitting allocated 75% of data (*n* = 112) for training and 25% (*n* = 38) for testing, maintaining proportional leakage-direction ratios to prevent sampling bias. Second, feature normalization scaled all input variables (e.g., *d*_50_, *A*_*leak*_) to a [0, 1] range using *MinMaxScaler* from Scikit-learn, addressing unit disparities and distribution skewness. Third, hyperparameter optimization employed *RandomizedSearchCV* (Scikit-learn) to evaluate 100 parameter combinations per model (Table 2), with optimization targeting RMSE minimization through 5-fold cross-validation on training data.

**Table 2:** Assigned values for the hyperparameters of each model.

Estimated parameter	Model		Hyperparameters (param_grid)
**H**_**f**_ ** /d**_**50**_	**Random Forest**		n_estimators': [50, 100, 150], 'max_depth': [5, 10, 15, 20], 'min_samples_split': [5, 10, 20], 'min_samples_leaf': [2, 5, 10], 'max_features': ['sqrt', 'log2']
	**XGBoost**		'n_estimators': [100, 200, 300], 'max_depth': [3, 4, 5], 'learning_rate': [0.01, 0.05, 0.1], 'subsample': [0.8, 0.9, 1.0], 'colsample_bytree': [0.8, 0.9, 1.0]
	**Stacking**	MLP model	'Hidden_layer_sizes': [(100,), (120,)], 'activation': ['relu', 'tanh'], 'solver': ['adam', 'sgd'], 'alpha': [0.0001, 0.001, 0.01,1], 'learning_rate': ['constant', 'adaptive'], 'learning_rate_init': [0.001, 0.01, 0.1]
		SVR model	'kernel': ['linear', 'poly', 'rbf', 'sigmoid'], 'C': [0.1, 1, 10, 100,1000], 'gamma': ['scale', 'auto'], 'degree': [2,3, 4, 5,6]
$\frac{\sqrt{A_{f}}}{d_{50}}$	**Random Forest**		'n_estimators': [50, 100, 150], 'max_depth': [5, 10, 15, 20], 'min_samples_split': [5, 10, 20], 'min_samples_leaf': [2, 5, 10], 'max_features': ['sqrt', 'log2']
	**XGBoost**		'n_estimators': [100, 200, 300], 'max_depth': [3, 4, 5], 'learning_rate': [0.01, 0.05, 0.1], 'subsample': [0.8, 0.9, 1.0], 'colsample_bytree': [0.8, 0.9, 1.0]
	**Stacking**	MLP model	'Hidden_layer_sizes': [(100,), (120,)], 'activation': ['relu', 'tanh'], 'solver': ['adam', 'sgd'], 'alpha': [0.0001, 0.001, 0.01,1], 'learning_rate': ['constant', 'adaptive'], 'learning_rate_init': [0.001, 0.01, 0.1]
		SVR model	'kernel': ['linear', 'poly', 'rbf', 'sigmoid'], 'C': [0.1, 1, 10, 100,1000], 'gamma': ['scale', 'auto'], 'degree': [2,3, 4, 5,6]

#### 2.5.3. Model evaluation and sensitivity analysis.

Model performance was comprehensively assessed using four metrics: the coefficient of determination (R^2^), root mean square error (RMSE), Taylor diagrams for visual comparison of R^2^, RMSE and correlation coefficients, and error distribution analysis to evaluate prediction consistency. Sensitivity analysis was conducted through leave-one-out (LOO) evaluation, which systematically ranked input parameter influence by measuring R^2^ reduction during iterative feature exclusion. Key parameters analyzed included soil uniformity coefficient (*Cu*), densimetric Froude number (Fr_d_), and critical geometric ratios such as *d*_50_/*A*_*leak*_.

## 3. Results and discussion

### 3.1. Fluidization zone mechanism over time

**Fig 5:**
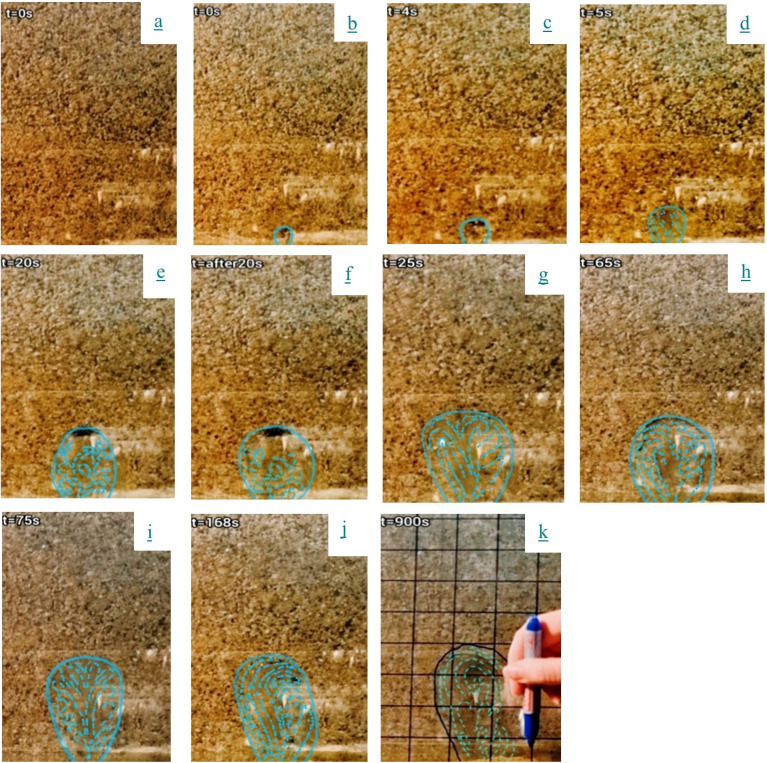
Time evolution of fluidization at *Q*_*leak*_ = 1.01E-04 m^3^/s: (a) Before the pump is activated, (b) At the start of the experiment, (c) At *t* = 4 s, (d) At *t* = 5 s, (e) At *t* = 20 s, (f) Immediately after *t *= 20 s, (g) At *t* = 25 s, (h) At *t *= 65 s, (i) At *t* = 75 s, (j) At *t* = 168 s, (k) At *t* = 900 s.

This section examines the temporal evolution of fluidization induced by a defined leakage discharge. By analyzing sequential video frames from the experimental process (Fig 5), seven distinct stages of development are identified. These stages reflect the coupled interaction between hydraulic and hydrodynamic forces, capturing the transition from initial jet penetration to the formation of a stable fluidized state. Understanding this progression is essential for evaluating soil stability under leak-induced jet flows and for modeling subsurface erosion mechanisms.

Fig 5 illustrates the fluidization dynamics observed for a representative leakage discharge rate of *Q*_*leak*_ = 1.01 × 10^−4^ m^3^/s. The process unfolds in seven primary stages, each governed by the interaction between internal pressure, seepage-induced stress, and particle mobilization:

**Stage 1: Initial cavitation (*t*** **=** **0–4** **s)** Pump activation generates a high-velocity jet that displaces soil particles via momentum transfer, forming a 2 cm cavity. This stage highlights the critical role of initial jet momentum in overcoming soil resistance. No sustained particulate transport occurs during this incubation phase (Fig 5a–c).

**Stage 2: Incipient rotation (*t* = 5 s)** Shear-induced turbulent vortices develop at the jet-soil interface, initiating rotational particle motion. This marks the onset of hydrodynamic forces dominating static friction thresholds, though the full fluidization regime remains unrealized (Fig 5d).

**Stage 3: Hydraulic fluidization (*t*** **=** **20** **s)** Elevated hydraulic gradients induce critical pore pressures, suspending particles through combined buoyancy and drag forces. The system transitions to a metastable fluid-like state with fountain flow patterns. This stage underscores the importance of pore pressure thresholds in facilitating particle mobilization (Fig 5e).

**Stage** **4: Quasi-equilibrium (*t*** = **20–25 s)** Particle migration decelerates as hydraulic energy partitioning creates a perched water reservoir above the cavity. Granulometric sorting emerges, with fines remaining suspended while coarser fractions undergo gravity-driven sedimentation. This phase highlights the role of hydraulic energy redistribution in stabilizing the fluidized zone (Fig 5f).

**Stage** **5: Vertical stabilization (*t*** **=** **25–65 s)** Cavity elongation occurs without lateral expansion, as particulate motion self-organizes into a characteristic fountain flow regime. Force equilibrium enables stable recirculation patterns, illustrating the balance between buoyancy, drag, and gravity (Fig 5g).

**Stage 6: Erosional instability (*t*** **=** **65–75** **s)** Jet impingement on upper cavity boundaries triggers renewed erosion, expanding the fluidized zone bidirectionally. Transient flow disorder precedes rapid reorganization, manifesting erosional propagation into overlying strata. This stage emphasizes the dynamic interplay between jet forces and soil erosion (Fig 5h).

**Stage 7:** **Terminal stabilization (*t*** > **75 s)** Dimensional stabilization occurs, though intra-zone kinematic variability persists. Vortex bifurcation alternates between single- and dual-celled structures, modulated by differential erosion susceptibility governed by particle-size distribution. In the fluidized zone, fine particles are more easily lifted and transported by the jet due to their low inertia, while coarser particles cause more rapid dissipation of jet momentum. This contrast in behavior creates spatial variability in flow intensity, which drives the formation of asymmetric or fluctuating vortex patterns. As a result, the heterogeneity of grain sizes plays a central role in influencing the symmetry, organization, and stability of flow structures in the final stage. This highlights the ongoing influence of both hydrodynamic forces and granular interactions on particle motion (Fig 5j–k).

This seven-stage progression reveals how hydraulic jets can induce time-dependent fluidization through a combination of mechanical displacement, pore pressure buildup, and force rebalancing. These findings not only enhance understanding of soil response under leak scenarios but also provide a framework for modeling jet-induced instability in granular media.

### 3.2. Effect of soil gradation and flow direction on fluidized zone dimensions

This section investigates the critical factors influencing fluidization development under varying subsurface conditions, focusing on the combined effects of soil gradation and flow direction on the geometry of fluidized zones. Understanding these relationships is essential for accurately predicting soil behavior during leakage events, which directly impacts subsurface erosion risk assessment and the design of mitigation strategies for buried infrastructure. To systematically analyze these effects, we utilized a comprehensive dataset comprising 150 controlled experimental trials. The dataset was categorized based on leakage direction—either upward or downward—and soil uniformity coefficient (*Cu*), resulting in three distinct groups:

1. Downward leakage with *Cu* less than 2 (*n* = 29),
2. Downward leakage with *Cu* greater than 2 (*n* = 40),
3. Upward leakage with *Cu* less than 2 (*n =* 81).

**Fig 6:**
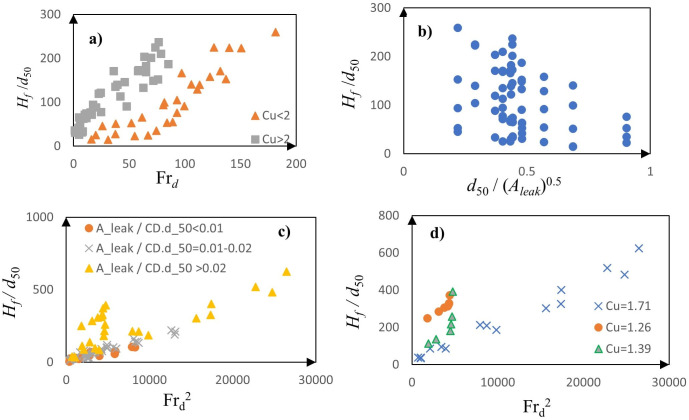
Variation of *H*_*f*_*/d*_50_ with: a) Fr_d_ for different *Cu* values in downward-directed leakage, (b) *d*_50_/√(*A*_*leak*_) in downward-directed leakage, (c) Fr_d_^2^ for different ranges of *A*_*leak*_*/(CDd*_50_) in upward-directed leakage, and (d) Fr_d_^2^ for different *Cu* values in upward-directed leakage.

For downward leakage, the dimensionless fluidization height (*H*_*f*_*/d*_50_) exhibited a near-linear relationship with the densimetric Froude number (Fr_*d*_), as shown in Fig 6a. This relationship, expressed as *H*_*f* _∝ *V*_*in*_/√ (*G*g*d*_50_), diverges from classical hydrodynamic theory, which typically predicts an inverse proportionality with particle size. This deviation is attributed to the synergistic interaction between downward jet momentum and gravity, where larger particle diameters create increased inter-granular voids. These voids facilitate the mobilization of finer particles through the coarse matrix under turbulent shear forces, intensifying fluidization.

Additionally, soils with higher uniformity coefficients (*Cu* > 2) exhibited a steeper trend in Fig 6a, indicating that grater soil gradation enhances fines migration and promotes vertical fluidization. The influence of leakage area (*A*_*leak*_) is also pivotal; Fig 6b shows a square-root scaling relationship *H*_*f*_*/d*_50_ ∝ √ (*A*_*leak*_), attributed to intensified turbulent eddies and localized shear stresses that promote particle suspension and upward transport. In contrast, upward leakage exhibited fundamentally different behavior. Here *H*_*f*_*/d*_50_ showed strong correlation with the squared densimetric Froude number (Fr_d_^2^), as illustrated in Fig 6c. This indicates that fluidization under upward flow is driven by kinetic energy transfer rather than linear momentum alone. Interestingly, in this configuration, soil non-uniformity (*Cu* > 2) inhibited fluidization (Fig 6d), likely due to the entrapment of fine particles within coarse-grain voids, which resist upward mobilization. Additionally, overburden pressure (represented by cover depth *CD*), further constrains vertical expansion, particularly at higher ratios of *A*_*leak*_*/*(*CDd*_50_). These results highlight a strong directional dependency in fluidization mechanisms: downward leakage synergizes with gravity to reinforce jet penetration, while upward leakage is limited by overburden resistance and mechanical interlocking of soil particles. Soil gradation plays a dual role: enhancing fluidization in downward flows but suppressing it under upward leakage due to fines entrapment and mechanical interlocking.

Further supporting this dual regime, Fig 6c compares fluidization heights for soils with varying *Cu* values (1.26, 1.39, and 1.71). Well-graded soils (*Cu* = 1.71) achieve significantly greater fluidized heights—approaching 700—indicating that once fluidization initiates, energy propagates deeper within these soils. Uniform soils, by contrast, show earlier fluidization onset but reach shallower fluidization depths, likely due to lower internal friction and reduced particle interlocking. This dual behavior is further evidenced in Fig 6a, which groups data into *Cu* < 2 and *Cu* > 2 categories. For a given Frd, well-graded soils consistently produce higher *H*_*f*_*/d*_50_ values. Uniform soils exhibit earlier fluidization onset but limited height, whereas graded soils delay onset but yield significantly greater fluidized depths once instability is triggered. This behavior likely stems from the higher internal friction and particle interlocking in graded soils, which initially resists fluidization but ultimately facilitates more extensive vertical deformation. In summary, the data reveal a dual behavioral regime: 1) Uniform soils (*Cu* < 2) facilitate early fluidization onset due to reduced particle interlocking, but their fluidized zones remain relatively shallow, and 2) Well-graded soils (Cu > 2), on the other hand, resist early fluidization but support significantly larger fluidized heights once hydraulic instability becomes dominant. These insights are crucial for predictive modeling and practical applications, especially in assessing subsurface erosion risks and designing mitigation strategies for buried pipeline infrastructure. By elucidating the contrasting roles of flow direction and soil gradation, this analysis advances the understanding necessary for accurately predicting fluidized zone geometry. The findings have direct implications for engineering practice, particularly in the design and risk assessment of buried pipelines and other subsurface infrastructure vulnerable to leakage-induced soil fluidization.

### 3.3. New predictive models for fluidized zone dimensions

Based on comprehensive dimensional analysis, empirical models were developed to predict the dimensionless maximum fluidized height (*H*_*f*_/*d*_50_) and normalized fluidized area (√*A*_*f*_*/d*_50_)) for both upward and downward leakage scenarios. The primary objective of this section is to translate experimental observations into generalized predictive equations that can inform engineering decisions under diverse soil and hydraulic conditions. Such models are particularly valuable for risk assessment in buried pipeline systems and for designing erosion mitigation strategies in cases where direct experimentation is impractical. The models reveal fundamentally different governing mechanisms between upward and downward leakage scenarios.

For downward leakage, the fluidized height follows:


$$\frac{H_{f}}{d_{\text{50}}}=\text{2}.\text{798}\;\;{{\text{Fr}}_{\text{d}}}^{\text{0}.\text{666}}{\left(\frac{d_{\text{50}}}{\sqrt{A_{leak}}}\right)}^{-\text{0}.\text{489}}{Cu}^{\text{0}.\text{493}}$$ (1)

while upward leakage exhibits distinct behavior:


$$\frac{H_{f}}{d_{50}}=6.803\;{\left({\text{Fr}}_{\text{d}}^{2}\right)}^{0.802}{\left(\frac{A_{leak}}{CDd_{50}}\right)}^{0.583}{Cu}^{-3.204}$$ (2)

The models demonstrate several critical features:

1. **Uniformity coefficient dominance**: *Cu*’s influence is markedly stronger in upward leakage (exponent −3.204) versus downward (0.493), reflecting eightfold greater sensitivity. This arises because downward leakage shows attenuated *Cu* dependence across broader soil gradations (*Cu* = 2–7.7), where localized decreasing trends diminish overall impact.
2. **Fluidization intensity**: The upward leakage coefficient (6.803) is 2.4 × larger than downwards (2.798), suggesting greater expansion potential – though soil cover depth (*CD*) in upward scenarios complicates direct comparison.
3. **Mechanistic drivers**: The analysis reveals fundamentally different behaviors between leakage directions based on soil uniformity coefficients (*Cu*). Downward leakage demonstrates beneficial dependence on *Cu* (positive exponent 0.493), where fines migration through coarse soil voids actively promotes fluidization. Conversely, upward leakage shows strong negative *Cu* dependence (exponent −3.204), indicating that overburden pressure significantly suppresses fines movement in this configuration.

**Fig 7:**
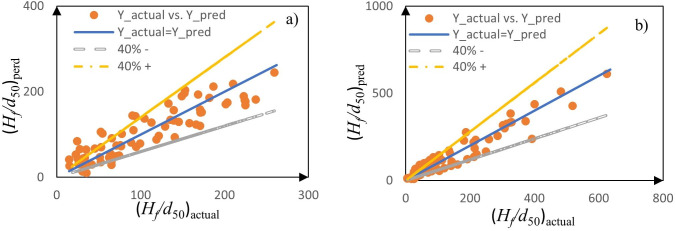
Density plot of observed and predicted data points from Eqs. (1) and (2) around the best-fit trend line ((*H*_*f*_/*d*_50_)_*perd*_ = (*H*_*f*_/*d*_50_)_*actual*_) for: a) downward-directed leakage, and b) upward-directed leakage.

**Fig 8:**
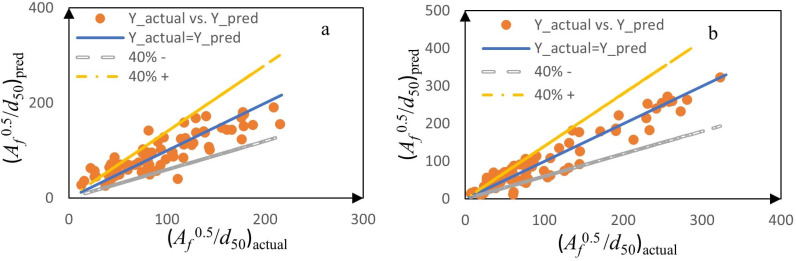
Density plot of observed and predicted data points from Eqs. (3) and (4) around the best fit trend line ((√*A*_*f*_/*d*_50_)_*perd*_ = (√*A*_*f*_/*d*_50_)_*actual*_) for: a) downward-directed leakage, and b) upward-directed leakage.

Model validation shows upward leakage predictions (R² = 0.915, RMSE = 37.789) outperform downward (R² = 0.792, RMSE = 29.937), primarily due to the latter’s dataset variability. However, both maintain practical utility with 71–75% of predictions within ±40% of experimental values (Figs 7 and 8).

The dimensionless area models maintain consistent parameter influences but show scaled coefficients:

For upward leakage:


$$\frac{\sqrt{A_{f}}}{d_{\text{50}}}{=\text{5}.\text{475}\left({\text{Fr}}_{\text{d}}\right)}^{0.414}{\left(\frac{d_{\text{50}}}{\sqrt{A_{leak}}}\right)}^{-\text{0}.\text{512}}{Cu}^{\text{0}.\text{626}}$$ (3)

For downward leakage:


$$\;\frac{\sqrt{A_{f}}}{d_{\text{50}}}{=\text{34}.\text{565}\left({{\text{Fr}}_{\text{d}}}^{\text{2}}\right)}^{\text{0}.\text{582}}{\left(\frac{A_{leak}}{CDd_{\text{50}}}\right)}^{\text{0}.\text{585}}{Cu}^{-\text{3}.\text{171}}$$ (4)

Key observations emerge from these relationships are:

1. **Parameter Consistency**: The exponent signs for all dimensionless parameters match those in the height prediction equations (Eqs. 1 and 2), confirming unified governing mechanisms for both height and area development. Notably, leakage area (*A*_*leak*_) maintains proportional relationships with both *H*_*f*_*/d*_50_ and ${\sqrt{A_{f}}/d}_{\text{50}}$.
2. **Coefficient Scaling**: For upward leakage, the area coefficient (34.565) is approximately five times greater than the corresponding height coefficient (6.803), indicating substantially different scaling relationships. In contrast, downward leakage demonstrates more moderate scaling behavior, with coefficients showing approximately a two-fold difference (5.475 versus 2.798). These distinct scaling ratios suggest fundamentally different physical mechanisms dominate in upward versus downward leakage scenarios.
3. **Geometric Relationships**: Simplified approximations reveal fundamental differences between leakage directions:

Upward leakage:


$$\frac{\sqrt{A_{f}}}{d_{\text{50}}}\approx \text{2}\frac{H_{f}}{d_{\text{50}}}\;\;\;\text{or}\;\;\;A_{f}\approx 4{H_{f}}^{2}$$ (5)

Downward leakage:


$$\frac{\sqrt{A_{f}}}{d_{\text{50}}}\approx 5\frac{H_{f}}{d_{\text{50}}}\;\;\;\text{or}\;\;\;\;A_{f}\approx 25{H_{f}}^{2}$$ (6)

These relationships demonstrate approximately 2.5 times greater fluidization intensity in downward leakage scenarios, a phenomenon driven by three key physical mechanisms. First, the absence of soil weight as a resistive force in downward leakage allows for more extensive fluidization development. Second, enhanced particle mobility occurs through increased void spaces between larger grains, facilitating finer particle movement. Third, the weight force acts additively to increase fluidization height rather than suppress it. The area prediction models demonstrate statistically significant performance across both leakage conditions, with upward leakage models achieving an R² of 0.912 (RMSE = 23.249) compared to downward leakage’s R² of 0.753 (RMSE = 24.840). This performance divergence primarily stems from the upward leakage dataset’s homogeneity (*Cu* < 2) versus the broader parameter range in downward leakage (*Cu* = 2–7.7). Interestingly, despite the lower R² values, downward leakage predictions exhibit tighter clustering, with 84% of data points falling within ±40% error bounds (Figs 8a and 8b) compared to 74% for upward leakage. This apparent contradiction between traditional regression metrics and prediction density underscores two critical methodological considerations: the substantial impact of parameter variability on regression statistics, and the essential practice of employing multiple evaluation criteria (including R², RMSE, and prediction bounds) for robust model assessment. The consistent physical relationships captured by both height and area models, combined with their demonstrated predictive capability across varying conditions, not only validate their practical utility for engineering applications but also highlight the fundamental mechanistic differences between upward and downward leakage scenarios.

### 3.4. Synthesis via Taylor diagrams

**Fig 9:**
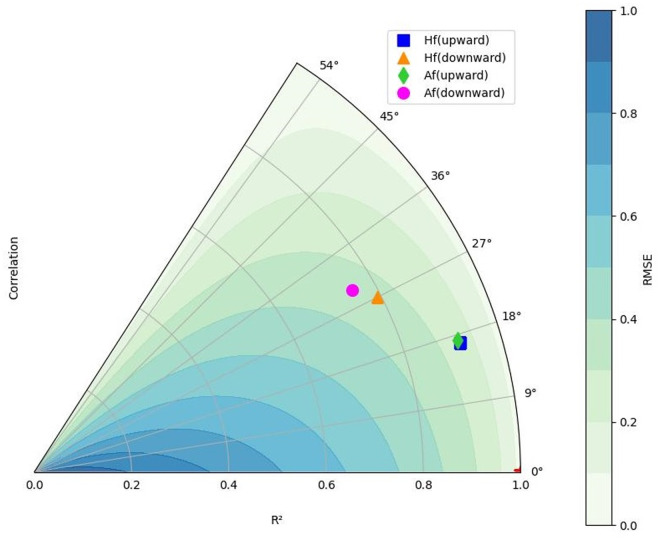
Taylor diagram based on R², RMSE, and correlation values for the equations of maximum height and area of the fluidized region.

As illustrated in Fig 9, the RMSE values for the height (*H*_*f*_*/d*_50_) and area (${\sqrt{A_{f}}/d}_{\text{50}}$) equations fall within similar ranges for each leakage direction:

- The triangular and circular markers, representing *H*_*f*_*/d*_50_ and ${\sqrt{A_{f}}/d}_{\text{50}}$ for downward-directed leakage, respectively, are located within the relative RMSE range of 0.3–0.4.
- The square and diamond-shaped markers, representing *H*_*f*_*/d*_50_ and ${\sqrt{A_{f}}/d}_{\text{50}}$ for upward-directed leakage, respectively, are positioned within the relative RMSE range of 0.2–0.3.

The performance of the area and height estimation equations is comparable in terms of RMSE for each leakage direction. This observation is further supported by similar trends in R² and correlation values. However, based on these evaluation metrics, the upward-directed leakage equations consistently demonstrate superior performance compared to the downward-directed leakage equations. Additionally, the height and area equations for upward-directed leakage exhibit greater similarity in performance, as evidenced by all three-evaluation metrics. In Fig 9, the diamond and square markers (representing *H*_*f*_*/d*_50_ and (√*A*_*f*_)/*d*_50_ for upward-directed leakage, respectively) are nearly overlapping and closer to the reference line, indicating higher predictive accuracy than the triangular and circular markers (representing downward-directed leakage). This difference in performance can be attributed to the greater variability in the downward-directed leakage dataset, particularly with respect to the uniformity coefficient (*Cu*). As previously discussed, downward-directed leakage encompasses a wider range of *Cu* values, whereas upward-directed leakage is limited to soils with *Cu* < 2.

### 3.5. Sensitivity analysis

This section employs a sensitivity analysis to prioritize the influential parameters in the equations *H*_*f*_*/d*_50_ and √(*A*_*f*_)/*d*_50_. The Leave-One-Out (LOO) method is used to quantify the relative importance of each parameter. The results, presented in Table 3, are summarized and analyzed below.

**Table 3:** Sensitivity analysis for $\frac{H_{f}}{d_{50}}\;$and $\frac{\sqrt{A_{f}}}{d_{50}}$ in upward-directed and downward-directed leakage.

Predicted paramete	Direction	Omitted parameter	Reduced percentage of R^2^	Resulted equation
$\frac{\sqrt{A_{f}}}{d_{\text{50}}}$	Upward	Cu	26.535	$\frac{\sqrt{A_{f}}}{d_{50}}=253.725{\left({{𝐅𝐫}_{d}}^{2}\right)}^{0.315}{\left(\frac{A_{leak}}{CD.d_{50}}\right)}^{0.870}$
		$\frac{A_{leak}}{CDd_{\text{50}}}$	9.211	$\frac{\sqrt{A_{f}}}{d_{50}}=0.672\;{\left({{𝐅𝐫}_{d}}^{2}\right)}^{0.833}{Cu}^{-4.496}$
		${{\text{Fr}}_{d}}^{\text{2}}$	34.320	$\frac{\sqrt{A_{f}}}{d_{50}}=19881.325{\left(\frac{A_{leak}}{CD.d_{50}}\right)}^{1.109}{Cu}^{-1.734}$
	Downward	Cu	71.315	$\frac{\sqrt{A_{f}}}{d_{50}}=38.055\;\;{{𝐅𝐫}_{d}}^{0.185}{\left(\frac{d_{50}}{\sqrt{A_{leak}}}\right)}^{-0.286}$
		$\frac{d_{\text{50}}}{\sqrt{A_{leak}}}$	8.632	$\frac{\sqrt{A_{f}}}{d_{50}}=8.908\;\;{{𝐅𝐫}_{d}}^{0.420}{Cu}^{0.581}$
		${{\text{Fr}}_{d}}^{\text{2}}$	68.526	$\frac{\sqrt{A_{f}}}{d_{50}}=35.408{\left(\frac{d_{50}}{\sqrt{A_{leak}}}\right)}^{-0.592}{Cu}^{0.344}$
$\frac{H_{f}}{d_{\text{50}}}$	Upward	Cu	15.956	$\frac{H_{f}}{d_{50}}=64.065\;\;{\left({{𝐅𝐫}_{d}}^{2}\right)}^{0.504}{\left(\frac{A_{leak}}{CD.d_{50}}\right)}^{0.853}$
		$\frac{A_{leak}}{CDd_{\text{50}}}$	6.667	$\frac{H_{f}}{d_{50}}=0.114\;{\left({{𝐅𝐫}_{d}}^{2}\right)}^{1.076}{Cu}^{-4.553}$
		${{\text{Fr}}_{d}}^{\text{2}}$	48.962	$\frac{H_{f}}{d_{50}}=44909.602\;{\left(\frac{A_{leak}}{CD.d_{50}}\right)}^{-0.987}{Cu}^{1.317}$
	Downward	Cu	34.596	$\frac{H_{f}}{d_{50}}=16.4\;\;{{𝐅𝐫}_{d}}^{0.415}\;{\left(\frac{d_{50}}{\sqrt{A_{leak}}}\right)}^{-0.357}$
		$\frac{d_{\text{50}}}{\sqrt{A_{leak}}}$	9.217	$\frac{H_{f}}{d_{50}}=3.392\;\;{{𝐅𝐫}_{d}}^{0.731}{Cu}^{0.466}$
		${{\text{Fr}}_{d}}^{\text{2}}$	84.091	$\frac{H_{f}}{d_{50}}=55.060\;{\left(\frac{d_{50}}{\sqrt{A_{leak}}}\right)}^{-0.652}{Cu}^{0.087}$

According to Table 3, the densimetric Froude number (Fr_d_) is the most influential parameter in both leakage directions. Specifically, for downward-directed leakage, removing Fr_d_ from the *H*_*f*_*/d*_50_ and √(*A*_*f*_)/*d*_50_ equations result in approximately 70% and 80% reductions in R^2^, respectively. This underscores the critical role of Fr_d_ in downward leakage dynamics. For upward-directed leakage, removing Fr_d_ decreases the R^2^ value of the *H*_*f*_*/d*_50_ and √(*A*_*f*_)/*d*_50_ equations by about 50% and 35%, respectively. Although these reductions are smaller than those observed in downward-directed leakage, Fr_d_ remains the most influential parameter in upward-directed leakage.

The uniformity coefficient (*Cu*) also plays a significant role, particularly in downward-directed leakage. In the √(*A*_*f*_)/*d*_50_ equation for downward-directed leakage, removing *Cu* results in the highest reduction in R² among all parameters, surpassing even Fr_d_. This highlights the importance of soil gradation variability in downward leakage. Additionally, *Cu* is identified as the second-most influential parameter in both *H*_*f*_*/d*_50_ and √(*A*_*f*_)/*d*_50_ equations across leakage directions, consistent with its strong correlation with fluidization behavior observed in earlier analyses. In contrast, dimensionless geometric parameters such as *A*_*leak*_/(*CDd*_50_) (upward-directed leakage) and *d*_50_/√ (*A*_*leak*_) (downward-directed leakage) exhibit single-digit percentage reductions in R² (5–10%) when removed. While these parameters rank last in terms of influence, their removal still leads to a measurable decline in model performance, indicating their non-negligible role.

### 3.6. Estimation of dimensionless fluidized zone parameters using Ensemble Learning

To enhance predictive capability beyond traditional empirical modeling, three ensemble machine learning approaches were implemented to estimate the dimensionless fluidized zone parameters—namely, the normalized height (*H*_*f*_*/d*_50_) and area (√(*A*_*f*_)/*d*_50_). The models employed were Random Forest (RF), eXtreme Gradient Boosting (XGBoost), and a Stacking ensemble that integrates multilayer perceptron (MLP) and support vector regression (SVR), with a linear regression model serving as the meta-learner.

The primary objective was to develop robust, data-driven models capable of capturing the nonlinear and multi-dimensional relationships governing fluidization dynamics. Unlike closed-form empirical equations, machine learning models can adapt to complex patterns and interactions among variables such as leakage discharge, orifice size, cover depth, and soil characteristics.

All models were trained using a dataset of 150 experimental observations and dimensionless input variables derived from the earlier dimensional analysis. Model development included thorough preprocessing, hyperparameter optimization, and evaluation based on key performance metrics. The models were rigorously optimized and evaluated to identify the most effective predictor for each geometric parameter.

#### 3.6.1. Hyperparameter optimization.

The randomized search cross-validation (*RandomizedSearchCV*) yielded optimal configurations for each model (Table 4), revealing several significant findings:

**Table 4:** Optimized hyperparameters of ensemble learning models.

Estimated parameter	Model	Chosen hyperparameters by *RandomizedSearchCV*	R^2^
$\frac{H_{f}}{d_{50}}$	Random Forest	: {*‘n_estimators’*: 50, *‘min_samples_split’*: 5, ‘min_samples_leaf’: 2, *‘max_features’*: ‘log2’, *‘max_depth’*: 20}	0.722
	XGBoost	{‘subsample’: 0.8, *‘n_estimators’*: 200, *‘max_depth’*: 3, *‘learning_rate’*: 0.05, *‘colsample_bytree’*: 0.8}	0.852
	Stacking	{‘solver’: ‘sgd’, *‘learning_rate_init’*: 0.001, *‘learning_rate’*: ‘constant’, *‘hidden_layer_sizes’*: (100,), ‘*alpha*’: 0.0001, ‘activation’: ‘tanh’}	0.901
		{‘*kernel*’: ‘rbf’, ‘*gamma*’: ‘scale’, ‘degree’: 2, ‘C’: 1000}	0.797
$\frac{\sqrt{A_{f}}}{d_{50}}$	Random Forest	{*‘n_estimators’*: 150, *‘min_samples_split’*: 5, *‘min_samples_leaf’*: 2, *‘max_features’*: ‘log2’, ‘max_depth’: 15}	0.732
	XGBoost	{‘subsample’: 0.8, *‘n_estimators’*: 300, *‘max_depth’*: 3, *‘learning_rate’*: 0.1, ‘colsample_bytree’: 0.9}	0.855
	Stacking	{‘solver’: ‘*adam*’, *‘learning_rate_init’*: 0.1, *‘learning_rate’*: ‘constant’, *‘hidden_layer_sizes’*: (100,), ‘*alpha*’: 0.0001, ‘activation’: ‘*relu*’}	0.873
		{‘kernel’: ‘*rbf*’, ‘*gamma*’: ‘scale’, ‘degree’: 2, ‘*C*’: 1000}	0.748

Random Forest Models: For the Random Forest model estimating *H*_*f*_*/d*_50_, the optimal number of estimators (trees) was determined to be 50. Since the initial values were 50, 100, and 150, selecting the smallest number suggests that increasing the number of trees beyond 50 may lead to overfitting or increased computational time without performance gains. In contrast, for √(*A*_*f*_)/*d*_50_, the optimal number of trees was 150, indicating that this model benefited from a larger ensemble.

The initial values for *max_depth* (tree depth) were 5, 10, 15, and 20. The selection of *max_depth* = 20 for *H*_*f*_*/d*_50_ and *max_depth* = 15 for √(*A*_*f*_)/*d*_50_ suggests two key points:

• The *H*_*f*_*/d*_50_ estimation model required learning more detailed features.
• Reducing the depth slightly for √(*A*_*f*_)/*d*_50_ helped balance bias and variance, improving generalization.

Additionally, *min_samples_split* = 5 and *min_samples_leaf* = 2 in both models indicate that preventing excessive *splits-maintained* model stability. The reduction of *max_features* to log2 in both models suggest that limiting the number of features per split reduced tree correlation, enhancing performance. The best R² values for the Random Forest models in estimating *H*_*f*_*/d*_50_ and √(*A*_*f*_)/*d*_50_ were similar.

XGBoost Models: For the XGBoost models, *max_depth* = 3 was selected for both *H*_*f*_*/d*_50_ and √(*A*_*f*_)/*d*_50_ indicating that deeper trees led to overfitting. The optimal configurations for the number of trees and learning rate were:

- For *H*_*f*_*/d*_50_: 200 trees with a learning rate of 0.05.
- For √(*A*_*f*_)/*d*_50_: 300 trees with a learning rate of 0.1.

This suggests that the √(*A*_*f*_)/*d*_50_ estimation model was more sensitive to additional iterations. For feature and data sampling (*colsample_bytree* and *subsample*), *subsample* = 0.8 was optimal for both models, while *colsample_bytree* = 0.9 for √(*A*_*f*_)/*d*_50_ outperformed *colsample_bytree* = 0.8 for *H*_*f*_*/d*_50_. The best R² score of approximately 0.85 for both XGBoost models indicates that XGBoost outperformed Random Forest in both cases.

Stacking Models: The Stacking model, combining MLP and SVR, achieved the highest performance, with a best R² score of nearly 0.9. This demonstrates the effectiveness of neural networks in estimating the geometric parameters of the fluidized region. Hyperparameter optimization revealed that:

- For *H*_*f*_*/d*_50_ estimation, the best configuration was *SGD + tanh*.
- For √(*A*_*f*_)/*d*_50_ estimation, the best configuration was *Adam + Relu*.

The SVR model within the Stacking ensemble exhibited the lowest performance, with a best R² score of approximately 0.75. To further explore the optimized hyperparameters, the corresponding heatmaps and the related explanations are provided in “S14 Heatmap”.

### 3.7. Model performance evaluation

**Fig 10:**
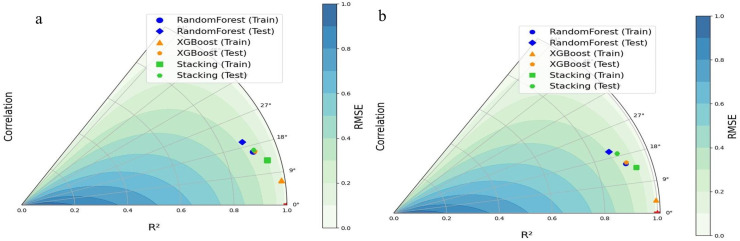
Taylor diagram for comparison and evaluation of ensemble learning models in estimating: a) *H*_*f*_*/d*_50_, and b) √(*A*_*f*_)/*d*_50._

Our comprehensive evaluation incorporating R² values, RMSE, and correlation coefficients (Fig 10 and Table 5) revealed distinct performance characteristics among the three modeling approaches.

**Table 5:** Evaluation metric (R², RMSE, and Correlation coefficient) in the train and test phases for ensemble learning models estimating *H*_*f*_*/d*_50_ and√(*A*_*f*_)/*d*_50._

Estimated parameter	Model	R^2^ train	R^2^ test	RMSE train	RMSE test	Train correlation	Test correlation
$\frac{H_{f}}{d_{50}}$	Random Forest	0.907	0.882	32.69	35.1	0.961	0.942
	XGBoost	0.985	0.916	12.87	29.59	0.993	0.964
	Stacking	0.95	0.912	23.45	32.49	0.975	0.959
$\frac{\sqrt{A_{f}}}{d_{50}}$	Random Forest	0.912	0.868	20.04	23.67	0.965	0.939
	XGBoost	0.997	0.913	3.55	19.27	0.999	0.966
	Stacking	0.945	0.896	15.96	20.18	0.973	0.948

For *H*_*f*_*/d*_50_ prediction, XGBoost demonstrated superior accuracy (R^2^ = 0.951 ± 0.034), though the stacked ensemble achieved moderate predictive performance (R^2^ = 0.931 ± 0.019) at greater computational cost. Random Forest models showed the best robustness but the weakest accuracy showing slightly inferior results (R^2^ = 0.895 ± 0.013). For √(*A*_*f*_)/*d*_50_ prediction XGBoost maintained strong accuracy (R^2^ = 0.955 ± 0.042) while preserving computational efficiency., where the stacked ensemble emerged as the moderate performer (R^2^ = 0.921 ± 0.024) in case of robustness, with Random Forest exhibited the lowest prediction variability (R^2^ = 0.89 ± 0.022), suggesting its generalization capability.

### 3.8. Stability and accuracy trade-offs in Ensemble Models

According to Figs 10(a, b), the triangular marker (representing the train phase of the XGBoost model) has the highest R^2^ among all models, indicating the greatest accuracy in the train phase (R^2 ^= 0.985 for estimating *H*_*f*_*/d*_50_ and 0.997 for estimating the √(*A*_*f*_)/*d*_50_), based on Table 5). However, the relatively large difference between the R^2^ values in the train and test phases (approximately 0.07) suggests that this model is more prone to overfitting compared to the others. This issue is also evident in Fig 10b. In other words, while XGBoost demonstrates high accuracy in estimating both *H*_*f*_*/d*_50_ and√(*A*_*f*_)/*d*_50_, it performs poorly in terms of stability. The Random Forest models have lower accuracy in estimating *H*_*f*_*/d*_50_ and √(*A*_*f*_)/*d*_50_ in terms of the R^2^ (approximately 0.9), but due to the small difference in the R^2^ between the train and test phases (approximately 0.025 for estimating *H*_*f*_*/d*_50_ and 0.04 for estimating the √(*A*_*f*_)/*d*_50_) the Random Forest model exhibits the best stability than other models. The Stacking models, which combine multilayer perceptron and support vector regression, achieve relatively moderate values for the coefficient of determination (approximately 0.95) with minimal differences between train and test phases (approximately 0.04 for both *H*_*f*_*/d*_50_ and√(*A*_*f*_)/*d*_50_), making them relatively-moderate models in terms of regression coefficient performance.

According to Fig 10 and Table 5, the Random Forest models exhibit the highest root mean square error values when estimating *H*_*f*_*/d*_50_ and √(*A*_*f*_)/*d*_50_ (RMSE = 33.895 ± 1.205 for estimating *H*_*f*_*/d*_50_ and RMSE = 21.855 ± 1.815 for estimating √(*A*_*f*_)/*d*_50_), making them the weakest models in terms of accuracy but the best in terms of stability based on the lowest differences in RMSE between train and test phases. Similar to the coefficient of determination analysis, XGBoost achieves the lowest RMSE values in both the train and test phases for estimating the *H*_*f*_*/d*_50_ and√(*A*_*f*_)/*d*_50_ (RMSE = 21.23 ± 8.36 for estimating *H*_*f*_*/d*_50_ and RMSE = 11.41 ± 7.86 for estimating √(*A*_*f*_)/*d*_50_). However, it still exhibits the highest difference between train and test RMSE values, reinforcing its weaker stability compared to other models. The Stacking model once again emerges as the moderately-performing model, with moderate RMSE values (RMSE = 27.97 ± 4.52 for *H*_*f*_*/d*_50_ and RMSE = 18.07 ± 2.11 for √(*A*_*f*_)/*d*_50_) and moderate difference in RMSE between the train and test phases. Following the same pattern as the R^2^ and RMSE, the Stacking model is identified as the moderately-performing model based on correlation coefficient values (Corr = 0.967 ± 0.008 for *H*_*f*_*/d*_50_ and Corr = 0.962 ± 0.013 for √(*A*_*f*_)/*d*_50_). It achieves the moderate accuracy in the train phase and the moderate difference between train and test correlation values, indicating relatively good stability. The Random Forest models exhibit the lowest correlation coefficient values when estimating *H*_*f*_*/d*_50_ and √(*A*_*f*_)/*d*_50_ (Corr = 0.952 ± 0.009 for estimating *H*_*f*_*/d*_50_ and Corr = 0.952 ± 0.0.013 for estimating √(*A*_*f*_)/*d*_50_), making them the weakest models in terms of accuracy but the best in terms of stability based on the lowest differences in RMSE between train and test phases. For *H*_*f*_*/d*_50_ and √(*A*_*f*_)/*d*_50_ prediction, XGBoost demonstrated superior accuracy (Corr = 0.979 ± 0.015 and Corr = 0.983 ± 0.017), though the weakest in term of stability.

When comparing the models for estimating the dimensionless maximum height and area of the fluidized region evaluation criteria lead to the same conclusions about which model performs better. Based on the evaluation criteria, the XGBoost model for estimating *H*_*f*_*/d*_50_ and √(*A*_*f*_)/*d*_50_ is considered better due to the highest accuracy and Random Forest model showed the best stability due to the lowest difference between train and test phases. However, the Random Forest model had the lowest accuracy in terms of all criteria. Based on the criteria, the Stacking models for estimating the dimensionless height and area of fluidized region have shown moderate performance both in accuracy and stability.

### 3.9. Comparison with empirical equations

The comparison between ensemble learning models and empirical equations (Table 6) reveals critical insights into their respective capabilities for predicting fluidized zone dimensions. For estimating *H*_*f*_*/d*_50_, the XGBoost model demonstrates superior overall performance in terms of R^2^, RMSE and correlation coefficient, while XGBoost emerges as the weakest option due to its low stability. This hierarchy is also evident for √(*A*_*f*_)/*d*_50_ estimation, where XGBoost achieves the highest accuracy in terms of all criteria despite known stability limitations.

**Table 6:** Evaluation metrices (R^2^, RMSE, and correlation coefficient) for the equations and the test phase of ensemble learning models.

Estimated parameter	Model	R^2^	RMSE	Correlation
$\frac{H_{f}}{d_{50}}$	Random Forest	0.882	35.1	0.942
	XGBoost	0.916	29.59	0.964
	Stacking	0.912	32.49	0.959
	Eq. (1)	0.792	29.937	0.89
	Eq. (2)	0.915	37.787	0.957
$\frac{\sqrt{A_{f}}}{d_{50}}$	Random Forest	0.868	23.67	0.939
	XGBoost	0.913	19.27	0.966
	Stacking	0.894	20.41	0.946
	Eq. (3)	0.753	24.84	0.868
	Eq. (4)	0.912	23.249	0.955

When comparing with empirical equations in terms of deterministic coefficient and correlation coefficient, Eqs. (2 and 4) shows accuracy comparable to XGBoost and even surpasses the Stacking and Random Forest models in *H*_*f*_*/d*_50_ and √(*A*_*f*_)/*d*_50_ estimation. When RMSE is prioritized, Eqs. (1 and 4) for *H*_*f*_*/d*_50_ and √(*A*_*f*_)/*d*_50_ in downward leakage scenarios exhibit comparable performance to XGBoost and lower RMSE than the RMSE of Random Forest model. For *H*_*f*_*/d*_50_ and √(*A*_*f*_)/*d*_50_ prediction, XGBoost outperforms empirical equations, but its substantial train-test performance gap raises concerns about reliability.

The choice of evaluation metric impacts model ranking. Considering R² values, the Eqs. (2 and 4) achieve comparable performance with ensemble learning models. Correlation coefficient analysis reinforces these trends. While in terms of RMSE values, Eqs. (1 and 4) for *H*_*f*_*/d*_50_ and √(*A*_*f*_)/*d*_50_ in downward leakage scenarios exhibit comparable performance to the ensemble learning models. Although XGBoost model achieves the best R², RMSE and correlation coefficient values, its poor stability ultimately renders the Stacking model the preferred ensemble approach, as it consistently balances high accuracy with robust generalization across both prediction tasks. This comprehensive analysis underscores that while empirical equations remain competitive in specific scenarios, ensemble methods generally offer superior performance for fluidized zone characterization.

## 4. Conclusion

This study investigated the evolution of fluidized zones under constant pressure conditions and predicted geometric characteristics of fully developed fluidized zone using dimensional analysis, sensitivity analysis, and ensemble machine learning. Empirical equations were formulated to estimate the dimensionless height (*H*_*f*_*/d*₅₀) and area (√*A*_*f*_*/d*₅₀) of the fluidized zone, and three ensemble models—Random Forest, XGBoost, and Stacking—were developed to enhance prediction accuracy.

A seven-stage fluidization mechanism was identified, influenced by jet momentum, pore pressure, and soil gradation. Dimensional and sensitivity analyses revealed the densimetric Froude number (Fr_*d*_), coefficient of uniformity (*Cu*), and geometry ratios (e.g., *d*₅₀/√*A*_leak_ for downward leakage and *A*_*leak*_*/(CDd*₅₀) for upward leakage) as the most influential parameters.

The empirical models for estimating *H*_*f*_*/d*_50_ achieved R² of 0.915 and RMSE of 37.79 for upward leakage, and R² of 0.792 and RMSE of 29.94 for downward leakage. The equations estimating √(*A*_*f*_)/*d*_50_ reach R² of 0.753 and RMSE of 24.84 for upward leakage, and R² of 0.912 and RMSE of 23.25 for downward leakage. The XGBoost model outperformed all other approaches regarding accuracy across the full dataset. Although Random Forest model had the lowest accuracy, outperformed other models in stability. Predictions by stacking ensemble learning model had shown moderate performance both in accuracy and stability.

These findings demonstrate that ensemble learning models offer substantial improvements in predicting fluidized zone geometry. Stacking model has been chosen as the preferred ensemble approach, as it consistently balances high accuracy with robust generalization across both prediction tasks. Such models can support more accurate assessments of internal erosion and sinkhole risk in geotechnical and hydraulic engineering applications, particularly under variable field conditions.

**Practical implications:**

- The proposed models allow reliable estimation of fluidized zone dimensions, aiding in the assessment and mitigation of internal erosion and sinkhole risks in civil and geotechnical engineering projects.
- The Stacking model, in particular, offers a robust predictive tool suitable for complex, variable field conditions.
- The methodology and results can support the design of safer hydraulic structures, early warning systems, and risk mapping for leakage-related ground instability.

Future research could expand on this work by incorporating additional variables, applying field-scale validation, and exploring advanced deep learning architectures to improve model generalizability.

## Supporting information

S1 Video High-frame-rate video recordings from which Visual data were extracted. (MP4)

S1 Dataset Input variables extracted including soil properties (*Cu, d*_*50*_*, CD, G*), leakage geometry (*A*_*leak*_), and flow characteristics (*V*_*inlet*_, calculated as *Q*_*leak*_/*A*_*leak*_) from studies [38–41]. (XLSX)

S2 Appendix Dimensional analysis. (DOCX)

S3 Dataset input data used as input for estimating √(*A*_*f*_)/*d*_50_ and *H_f_*/*d*_50_ curve-fit. (XLSX)

S4 Dataset input data used as input for predicting √(*A*_*f*_)/*d*_50_ and *H_f_*/*d*_50_ by ensemble-machine learning methods. (XLSX)

S4 Code Source code for equation estimating √(A_f_)/d_50_ in downward leakage direction. (IPYNB)

S5 Code Source code for equation estimating √(A_f_)/d_50_ in upward leakage direction. (IPYNB)

S6 Code Source code for equation estimating H_f_/d_50_ in downward leakage direction. (IPYNB)

S7 Code Source code for equation estimating H_f_/d_50_ in upward leakage direction. (IPYNB)

S8 Code Source code for predicting √(A_f_)/d_50_ by Random Forest model. (IPYNB)

S9 Code Source code for predicting H_f_/d_50_ by Random Forest model. (IPYNB)

S10 Code Source code for predicting √(A_f_)/d_50_ by XGBoost model. (IPYNB)

S11 Code Source code for predicting H_f_/d_50_ by XGBoost model. (IPYNB)

S12 Code Source code for predicting √(A_f_)/d_50_ by Stacking model. (IPYNB)

S13 Code Source code for predicting H_f_/d_50_ by Stacking model. (IPYNB)

S14 Heatmap Hyperparameter Optimization via Heatmap Analysis. (PDF)
