# Supplementary material for: Experimental characterization and machine learning modeling of leakage-induced soil fluidization in water distribution systems
Source: PLoS One. 2025 Sep 23;20(9):e0331097. doi: 10.1371/journal.pone.0331097 (PMC12456785; doi:10.1371/journal.pone.0331097)
Supplement: S2 Appendix — (DOCX) [file pone.0331097.s003.docx]

**Data used for upward-directed leakage**

| No. | Author | *A_leak_*  (m^2^) | *G* | *CD*  (m) | *d*_50_  (m) | *Cu* | Pressure  (bar) | *Q_leak_*  (m^3^/s) | *H_f_*  (m) | *A_f_*  (m^2^) |  |
| --- | --- | --- | --- | --- | --- | --- | --- | --- | --- | --- | --- |
| 1 | Present research | 7.065E-06 | 2.7 | 0.485 | 3.304E-03 | 1.548 | 1.5 | 4.28E-05 | 8.500E-02 | 3.976E-02 |  |
| 2 |  |  |  |  |  |  | 2.5 | 6.04E-05 | 1.000E-01 | 4.081E-02 |  |
| 3 |  |  |  |  |  |  | 3.5 | 6.42E-05 | 9.500E-02 | 5.470E-03 |  |
| 4 |  |  |  |  | 1.606E-03 | 1.537 | 1.5 | 4.82E-05 | 5.000E-02 | 1.429E-03 |  |
| 5 |  |  |  |  |  |  | 2.5 | 7.51E-05 | 9.000E-02 | 2.408E-03 |  |
| 6 |  |  |  |  |  |  | 3.5 | 9.22E-05 | 7.000E-02 | 3.310E-03 |  |
| 7 |  |  |  |  | 1.358E-03 | 1.665 | 1.5 | 6.96E-05 | 4.000E-02 | 1.519E-03 |  |
| 8 |  |  |  | 0.372 |  |  | 2.5 | 6.22E-05 | 1.100E-01 | 8.420E-03 |  |
| 9 |  |  |  |  |  |  | 3.5 | 1.01E-04 | 1.400E-01 | 1.311E-02 |  |
| 10 |  |  |  |  | 1.358E-03 | 1.665 | 1.5 | 7.04E-05 | 4.000E-02 | 1.383E-03 |  |
| 11 |  |  |  |  |  |  | 2.5 | 7.56E-05 | 8.000E-02 | 4.765E-03 |  |
| 12 |  |  |  |  |  |  | 3.5 | 8.71E-05 | 1.240E-01 | 1.098E-02 |  |
| 13 |  |  |  |  | 1.606E-03 | 1.537 | 1.5 | 5.88E-05 | 4.100E-02 | 1.885E-03 |  |
| 14 |  |  |  |  |  |  | 2.5 | 7.14E-05 | 9.500E-02 | 6.556E-03 |  |
| 15 |  |  |  |  |  |  | 3.5 | 8.14E-05 | 1.000E-01 | 6.635E-03 |  |
| 16 |  |  |  |  | 3.304E-03 | 1.548 | 1.5 | 7.51E-05 | 1.200E-01 | 8.930E-03 |  |
| 17 | Hamedi  (2020) | 8.500E-06 | 2.65 | 0.4 | 6.400E-04 | 1.710 | 1.5 | 3.52E-05 | 2.390E-02 | 5.890E-04 |  |
| 18 |  |  |  |  |  |  | 2.5 | 6.90E-05 | 5.500E-02 | 2.372E-03 |  |
| 19 |  |  |  |  |  |  | 3.5 | 1.09E-04 | 1.196E-01 | 7.547E-03 |  |
| 20 |  |  |  |  |  |  | 4.5 | 1.45E-04 | 2.567E-01 | 2.183E-02 |  |
| 21 |  |  |  |  |  |  | 5.5 | 1.78E-04 | 4.000E-01 | 4.270E-02 |  |
| 22 |  |  |  |  | 1.260E-03 | 1.950 | 1.5 | 3.85E-05 | 2.890E-02 | 8.950E-04 |  |
| 23 |  |  |  |  |  |  | 2.5 | 7.90E-05 | 4.130E-02 | 1.753E-03 |  |
| 24 |  |  |  |  |  |  | 3.5 | 1.08E-04 | 1.509E-01 | 9.962E-03 |  |
| 25 |  |  |  |  |  |  | 4.5 | 1.42E-04 | 1.805E-01 | 1.572E-02 |  |
| 26 |  |  |  |  |  |  | 5.5 | 1.73E-04 | 2.775E-01 | 3.333E-02 |  |
| 27 |  |  |  |  | 2.000E-03 | 1.570 | 1.5 | 3.95E-05 | 2.260E-02 | 5.860E-04 |  |
| 28 |  |  |  |  |  |  | 2.5 | 7.93E-05 | 1.380E-01 | 6.406E-03 |  |
| 29 |  |  |  |  |  |  | 3.5 | 1.09E-04 | 1.520E-01 | 1.496E-02 |  |
| 30 |  |  |  |  |  |  | 4.5 | 1.50E-04 | 1.873E-01 | 2.358E-02 |  |
| 31 |  |  |  |  |  |  | 5.5 | 1.81E-04 | 2.685E-01 | 3.243E-02 |  |
| 32 |  |  |  | 0.5 | 6.400E-04 | 1.710 | 1.5 | 3.51E-05 | 2.220E-02 | 6.530E-04 |  |
| 33 |  |  |  |  |  |  | 2.5 | 6.44E-05 | 6.150E-02 | 2.696E-03 |  |
| 34 |  |  |  |  |  |  | 3.5 | 1.02E-04 | 1.348E-01 | 7.093E-03 |  |
| 35 |  |  |  |  |  |  | 4.5 | 1.45E-04 | 2.085E-01 | 1.546E-02 |  |
| 36 |  |  |  |  |  |  | 5.5 | 1.73E-04 | 3.090E-01 | 2.690E-02 |  |
| 37 |  |  |  |  | 1.260E-03 | 1.950 | 1.5 | 3.63E-05 | 2.580E-02 | 7.920E-04 |  |
| 38 |  |  |  |  |  |  | 2.5 | 7.65E-05 | 4.060E-02 | 1.798E-03 |  |
| 39 |  |  |  |  |  |  | 3.5 | 1.08E-04 | 1.232E-01 | 8.232E-03 |  |
| 40 |  |  |  |  |  |  | 4.5 | 1.38E-04 | 2.031E-01 | 1.844E-02 |  |
| 41 |  |  |  |  |  |  | 5.5 | 1.76E-04 | 2.669E-01 | 2.719E-02 |  |
| 42 |  |  |  |  | 2.000E-03 | 1.570 | 1.5 | 3.84E-05 | 1.030E-02 | 2.550E-04 |  |
| 43 |  |  |  |  |  |  | 2.5 | 7.89E-05 | 6.740E-02 | 4.010E-03 |  |
| 44 |  |  |  |  |  |  | 3.5 | 1.04E-04 | 1.429E-01 | 1.309E-02 |  |
| 45 |  |  |  |  |  |  | 4.5 | 1.49E-04 | 1.523E-01 | 1.426E-02 |  |
| 46 |  |  |  |  |  |  | 5.5 | 1.76E-04 | 2.076E-01 | 2.768E-02 |  |
| 47 |  |  |  | 0.6 | 6.400E-04 | 1.710 | 1.5 | 2.93E-05 | 2.440E-02 | 5.390E-04 |  |
| 48 |  |  |  |  |  |  | 2.5 | 5.04E-05 | 5.580E-02 | 2.345E-03 |  |
| 49 |  |  |  |  |  |  | 3.5 | 9.78E-05 | 1.368E-01 | 8.637E-03 |  |
| 50 |  |  |  |  |  |  | 4.5 | 1.37E-04 | 1.937E-01 | 1.489E-02 |  |
| 51 |  |  |  |  |  |  | 5.5 | 1.65E-04 | 3.323E-01 | 3.045E-02 |  |
| 52 |  |  |  |  | 1.260E-03 | 1.950 | 1.5 | 3.48E-05 | 2.340E-02 | 5.420E-04 |  |
| 53 |  |  |  |  |  |  | 2.5 | 7.65E-05 | 5.780E-02 | 2.327E-03 |  |
| 54 |  |  |  |  |  |  | 3.5 | 1.08E-04 | 1.314E-01 | 9.350E-03 |  |
| 55 |  |  |  |  |  |  | 4.5 | 1.40E-04 | 1.776E-01 | 1.724E-02 |  |
| 56 |  |  |  |  |  |  | 5.5 | 1.76E-04 | 2.415E-01 | 2.488E-02 |  |
| 57 |  |  |  |  | 2.000E-03 | 1.570 | 1.5 | 3.77E-05 | 9.100E-03 | 1.880E-04 |  |
| 58 |  |  |  |  |  |  | 2.5 | 7.52E-05 | 4.810E-02 | 2.381E-03 |  |
| 59 |  |  |  |  |  |  | 3.5 | 1.02E-04 | 9.340E-02 | 7.996E-03 |  |
| 60 |  |  |  |  |  |  | 4.5 | 1.48E-04 | 1.134E-01 | 9.854E-03 |  |
| 61 |  |  |  |  |  |  | 5.5 | 1.72E-04 | 2.136E-01 | 2.565E-02 |  |
| 62 | Mohammadbeigi  (2019) | 8.000E-06 | 2.65 | 0.5 | 6.100E-04 | 1.257 | 2.0 | 4.30E-05 | 1.524E-01 | 1.686E-02 |  |
| 63 |  |  |  |  |  |  | 2.5 | 5.62E-05 | 1.740E-01 | 1.959E-02 |  |
| 64 |  |  |  |  |  |  | 3.0 | 6.20E-05 | 1.874E-01 | 2.177E-02 |  |
| 65 |  |  |  |  |  |  | 3.5 | 6.52E-05 | 1.912E-01 | 2.309E-02 |  |
| 66 |  |  |  |  |  |  | 4.0 | 6.60E-05 | 1.983E-01 | 2.480E-02 |  |
| 67 |  |  |  |  |  |  | 4.5 | 6.64E-05 | 2.007E-01 | 2.560E-02 |  |
| 68 |  |  |  |  |  |  | 5.0 | 6.70E-05 | 2.274E-01 | 2.934E-02 |  |
| 69 |  |  |  |  | 6.900E-04 | 1.396 | 2.0 | 4.70E-05 | 7.780E-02 | 5.520E-03 |  |
| 70 |  |  |  |  |  |  | 2.5 | 5.68E-05 | 9.460E-02 | 6.732E-03 |  |
| 71 |  |  |  |  |  |  | 3.5 | 7.19E-05 | 1.249E-01 | 9.910E-03 |  |
| 72 |  |  |  |  |  |  | 4.0 | 7.25E-05 | 1.497E-01 | 1.391E-02 |  |
| 73 |  |  |  |  |  |  | 4.5 | 7.32E-05 | 1.784E-01 | 1.737E-02 |  |
| 74 |  |  |  |  |  |  | 5.0 | 7.38E-05 | 2.710E-01 | 2.596E-02 |  |
| 75 |  |  |  |  | 1.146E-03 | 1.551 | 2.0 | 5.08E-05 | 4.670E-02 | 1.662E-03 |  |
| 76 |  |  |  |  |  |  | 2.5 | 6.71E-05 | 5.230E-02 | 2.656E-03 |  |
| 77 |  |  |  |  |  |  | 3.0 | 7.31E-05 | 6.380E-02 | 3.714E-03 |  |
| 78 |  |  |  |  |  |  | 3.5 | 8.08E-05 | 7.670E-02 | 4.510E-03 |  |
| 79 |  |  |  |  |  |  | 4.0 | 8.71E-05 | 8.210E-02 | 4.984E-03 |  |
| 80 |  |  |  |  |  |  | 4.5 | 9.05E-05 | 8.690E-02 | 6.320E-03 |  |
| 81 |  |  |  |  |  |  | 5.0 | 9.65E-05 | 1.132E-01 | 9.822E-03 |  |

**Data used for downward-directed leakage**

| No. | Author | *A_leak_*  (m^2^) | *G* | *CD*  (m) | *d*_50_  (m) | *Cu* | Pressure  (bar) | *Q_leak_*  (m^3^/s) | *H_f_*  (m) | *A_f_*  (m^2^) |
| --- | --- | --- | --- | --- | --- | --- | --- | --- | --- | --- |
| 82 | Lessan  (2021) | 8.500E-06 | 2.65 | 0.12 | 6.400E-04 | 1.72 | 1.5 | 2.851E-05 | 2.910E-02 | 7.000E-04 |
| 83 |  |  |  |  |  |  | 2.5 | 5.684E-05 | 3.370E-02 | 8.870E-04 |
| 84 |  |  |  |  |  |  | 3.5 | 8.838E-05 | 6.000E-02 | 2.321E-03 |
| 85 |  |  |  |  |  |  | 4.5 | 1.501E-04 | 9.780E-02 | 3.813E-03 |
| 86 |  |  |  |  |  |  | 5.5 | 1.990E-04 | 1.660E-01 | 1.061E-02 |
| 87 |  |  |  |  | 1.260E-03 | 1.96 | 1.5 | 3.087E-05 | 3.170E-02 | 1.094E-03 |
| 88 |  |  |  |  |  |  | 2.5 | 5.802E-05 | 3.450E-02 | 1.078E-03 |
| 89 |  |  |  |  |  |  | 3.5 | 9.454E-05 | 8.270E-02 | 4.338E-03 |
| 90 |  |  |  |  |  |  | 4.5 | 1.493E-04 | 2.093E-01 | 2.161E-02 |
| 91 |  |  |  |  |  |  | 5.5 | 2.027E-04 | 2.158E-01 | 1.770E-02 |
| 92 |  |  |  |  | 2.000E-03 | 1.57 | 1.5 | 3.120E-05 | 3.050E-02 | 6.390E-04 |
| 93 |  |  |  |  |  |  | 2.5 | 6.022E-05 | 2.950E-02 | 9.910E-04 |
| 94 |  |  |  |  |  |  | 3.5 | 7.325E-05 | 1.027E-01 | 7.353E-03 |
| 95 |  |  |  |  |  |  | 4.5 | 1.584E-04 | 1.984E-01 | 2.198E-02 |
| 96 |  |  |  |  |  |  | 5.5 | 2.054E-04 | 2.817E-01 | 2.531E-02 |
| 97 |  | 4.906E-06 |  |  | 6.400E-04 | 1.72 | 1.5 | 5.877E-05 | 6.690E-02 | 2.304E-03 |
| 98 |  |  |  |  |  |  | 2.5 | 7.168E-05 | 8.940E-02 | 3.618E-03 |
| 99 |  |  |  |  |  |  | 3.5 | 7.978E-05 | 1.440E-01 | 8.356E-03 |
| 100 |  |  |  |  |  |  | 4.5 | 8.855E-05 | 1.433E-01 | 6.436E-03 |
| 101 |  |  |  |  |  |  | 5.5 | 9.529E-05 | 1.427E-01 | 6.590E-03 |
| 102 |  |  |  |  | 1.260E-03 | 1.96 | 1.5 | 5.937E-05 | 3.070E-02 | 7.770E-04 |
| 103 |  |  |  |  |  |  | 2.5 | 7.898E-05 | 6.870E-02 | 2.938E-03 |
| 104 |  |  |  |  |  |  | 3.5 | 8.849E-05 | 1.154E-01 | 8.048E-03 |
| 105 |  |  |  |  |  |  | 4.5 | 9.885E-05 | 1.626E-01 | 1.029E-02 |
| 106 |  |  |  |  |  |  | 5.5 | 1.089E-04 | 1.994E-01 | 1.551E-02 |
| 107 |  |  |  |  | 2.000E-03 | 1.57 | 1.5 | 6.139E-05 | 4.550E-02 | 4.930E-02 |
| 108 |  |  |  |  |  |  | 2.5 | 8.332E-05 | 7.010E-02 | 3.107E-03 |
| 109 |  |  |  |  |  |  | 3.5 | 9.394E-05 | 1.060E-01 | 6.584E-03 |
| 110 |  |  |  |  |  |  | 4.5 | 1.041E-04 | 1.521E-01 | 1.056E-02 |
| 111 | Sharafodin  (2022) | 7.000E-06 | 2.65 | 0.9 | 1.270E-03 | 7.7 | 1.5 | 1.806E-06 | 3.911E-02 | 2.571E-03 |
| 112 |  |  |  |  |  |  | 2.5 | 5.722E-06 | 5.326E-02 | 4.159E-03 |
| 113 |  |  |  |  |  |  | 3.5 | 7.500E-06 | 6.693E-02 | 5.789E-03 |
| 114 |  |  |  |  |  |  | 4.5 | 1.114E-05 | 7.920E-02 | 7.336E-03 |
| 115 |  |  |  |  |  |  | 5.5 | 1.461E-05 | 8.127E-02 | 9.664E-03 |
| 116 |  | 8.500E-06 |  |  |  |  | 1.5 | 1.172E-05 | 3.933E-02 | 5.954E-03 |
| 117 |  |  |  |  |  |  | 2.5 | 2.542E-05 | 9.038E-02 | 1.148E-02 |
| 118 |  |  |  |  |  |  | 3.5 | 3.778E-05 | 1.546E-01 | 2.215E-02 |
| 119 |  |  |  |  |  |  | 4.5 | 6.106E-05 | 1.771E-01 | 3.105E-02 |
| 120 |  |  |  |  |  |  | 5.5 | 1.006E-04 | 2.312E-01 | 4.969E-02 |
| 121 |  | 1.000E-05 |  |  |  |  | 1.5 | 2.731E-05 | 9.738E-02 | 1.034E-02 |
| 122 |  |  |  |  |  |  | 2.5 | 4.517E-05 | 9.850E-02 | 1.323E-02 |
| 123 |  |  |  |  |  |  | 3.5 | 6.850E-05 | 1.720E-01 | 3.038E-02 |
| 124 |  |  |  |  |  |  | 4.5 | 1.186E-04 | 2.138E-01 | 5.150E-02 |
| 125 |  |  |  |  |  |  | 5.5 | 1.426E-04 | 2.668E-01 | 7.018E-02 |
| 126 |  | 7.065E-06 |  |  |  |  | 1.5 | 6.122E-05 | 1.148E-01 | 1.061E-02 |
| 127 |  |  |  |  |  |  | 2.5 | 8.039E-05 | 1.689E-01 | 2.225E-02 |
| 128 |  |  |  |  |  |  | 3.5 | 9.217E-05 | 1.893E-01 | 2.700E-02 |
| 129 |  |  |  |  |  |  | 4.5 | 9.736E-05 | 1.934E-01 | 3.162E-02 |
| 130 |  |  |  |  |  |  | 5.5 | 1.092E-04 | 2.373E-01 | 5.075E-02 |
| 131 |  | 7.000E-06 |  |  | 1.170E-03 | 6 | 1.5 | 1.278E-06 | 4.167E-02 | 1.877E-03 |
| 132 |  |  |  |  |  |  | 2.5 | 5.083E-06 | 3.912E-02 | 2.995E-03 |
| 133 |  |  |  |  |  |  | 3.5 | 6.056E-06 | 7.619E-02 | 9.283E-03 |
| 134 |  |  |  |  |  |  | 4.5 | 8.445E-06 | 7.619E-02 | 1.089E-02 |
| 135 |  |  |  |  |  |  | 5.5 | 1.403E-05 | 8.690E-02 | 1.194E-02 |
| 136 |  | 8.500E-06 |  |  |  |  | 1.5 | 7.306E-06 | 3.018E-02 | 2.406E-03 |
| 137 |  |  |  |  |  |  | 2.5 | 1.483E-05 | 8.333E-02 | 9.182E-03 |
| 138 |  |  |  |  |  |  | 3.5 | 2.600E-05 | 1.107E-01 | 1.550E-02 |
| 139 |  |  |  |  |  |  | 4.5 | 6.239E-05 | 1.321E-01 | 1.933E-02 |
| 140 |  |  |  |  |  |  | 5.5 | 8.600E-05 | 2.000E-01 | 3.769E-02 |
| 141 |  | 1.000E-05 |  |  |  |  | 1.5 | 1.464E-05 | 3.929E-02 | 3.249E-03 |
| 142 |  |  |  |  |  |  | 2.5 | 3.508E-05 | 1.036E-01 | 1.507E-02 |
| 143 |  |  |  |  |  |  | 3.5 | 3.975E-05 | 1.393E-01 | 2.818E-02 |
| 144 |  |  |  |  |  |  | 4.5 | 6.300E-05 | 2.000E-01 | 4.243E-02 |
| 145 |  |  |  |  |  |  | 5.5 | 1.108E-04 | 2.381E-01 | 6.345E-02 |
| 146 |  | 7.065E-06 |  |  |  |  | 1.5 | 5.669E-05 | 1.702E-01 | 1.782E-02 |
| 147 |  |  |  |  |  |  | 2.5 | 7.211E-05 | 2.024E-01 | 2.300E-02 |
| 148 |  |  |  |  |  |  | 3.5 | 8.595E-05 | 2.357E-01 | 3.319E-02 |
| 149 |  |  |  |  |  |  | 4.5 | 9.072E-05 | 2.631E-01 | 4.355E-02 |
| 150 |  |  |  |  |  |  | 5.5 | 9.428E-05 | 2.774E-01 | 4.808E-02 |

**2.3. Dimensional analysis**

Dimensional analysis was conducted to identify the key factors influencing the fluidization phenomenon. Based on Buckingham’s Pi theorem, the following dimensionless parameters were derived:

Upward leakage:

| (1) | $\frac{H_{f}}{d_{50}}=f(\mathrm{Fr}_{d}, \frac{A_{leak}}{CDd_{50}},Cu)$ |
| --- | --- |
| (2) | $\frac{\sqrt{A_{f}}}{d_{50}}=f(\mathrm{Fr}_{d}, \frac{A_{leak}}{CDd_{50}},Cu)$ |

Downward leakage:

| (3) | $\frac{H_{f}}{d_{50}}=f(\mathrm{Fr}_{d},\frac{d_{50}}{\sqrt{A_{leak}}},Cu)$ |
| --- | --- |
| (4) | $\frac{\sqrt{A_{f}}}{d_{50}}=f(\mathrm{Fr}_{d}, \frac{d_{50}}{\sqrt{A_{leak}}},Cu)$ |

here, Fr_d_​ represents the densimetric Froude number, calculated as:

| (5) | $\mathrm{Fr}_{d}=\frac{V_{inlet}}{\sqrt{gGd_{50}}}$ |
| --- | --- |

Empirical relationships were derived using Python programming, with curve fitting performed using the SciPy library. The accuracy of the models was evaluated using the coefficient of determination (R²), root mean square error (RMSE), and Taylor diagrams. Sensitivity analysis was conducted using the Leave-One-Out method to assess the influence of each input parameter.
