# Supplementary material for: Experimental characterization and machine learning modeling of leakage-induced soil fluidization in water distribution systems
Source: PLoS One. 2025 Sep 23;20(9):e0331097. doi: 10.1371/journal.pone.0331097 (PMC12456785; doi:10.1371/journal.pone.0331097)
Supplement: S14 Heatmap — (PDF) [file pone.0331097.s016.pdf]

hyperparameter heatmap for RandomForest model predicting dimensionless a)A_f , b)H_f of fluidized zone

a

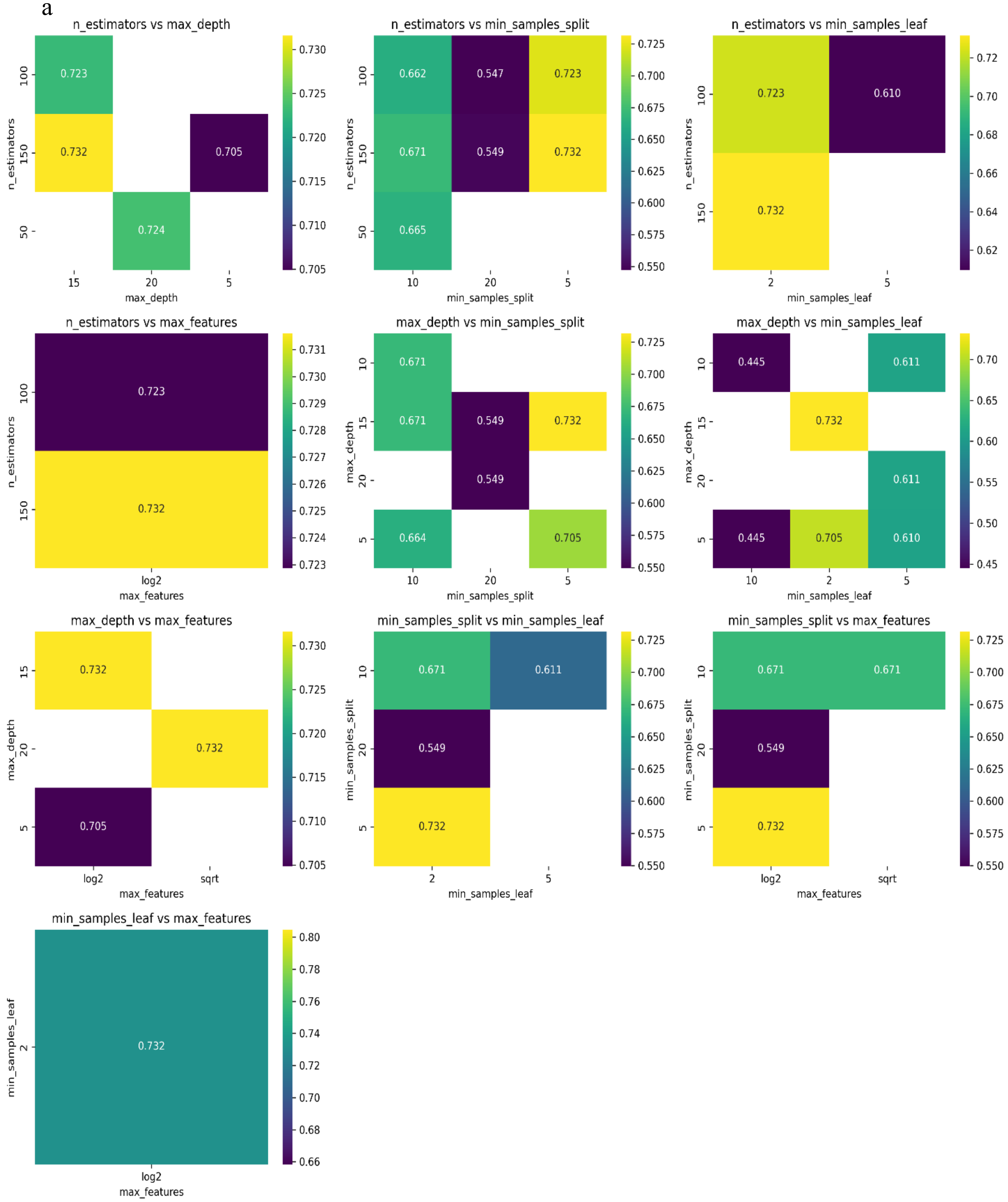

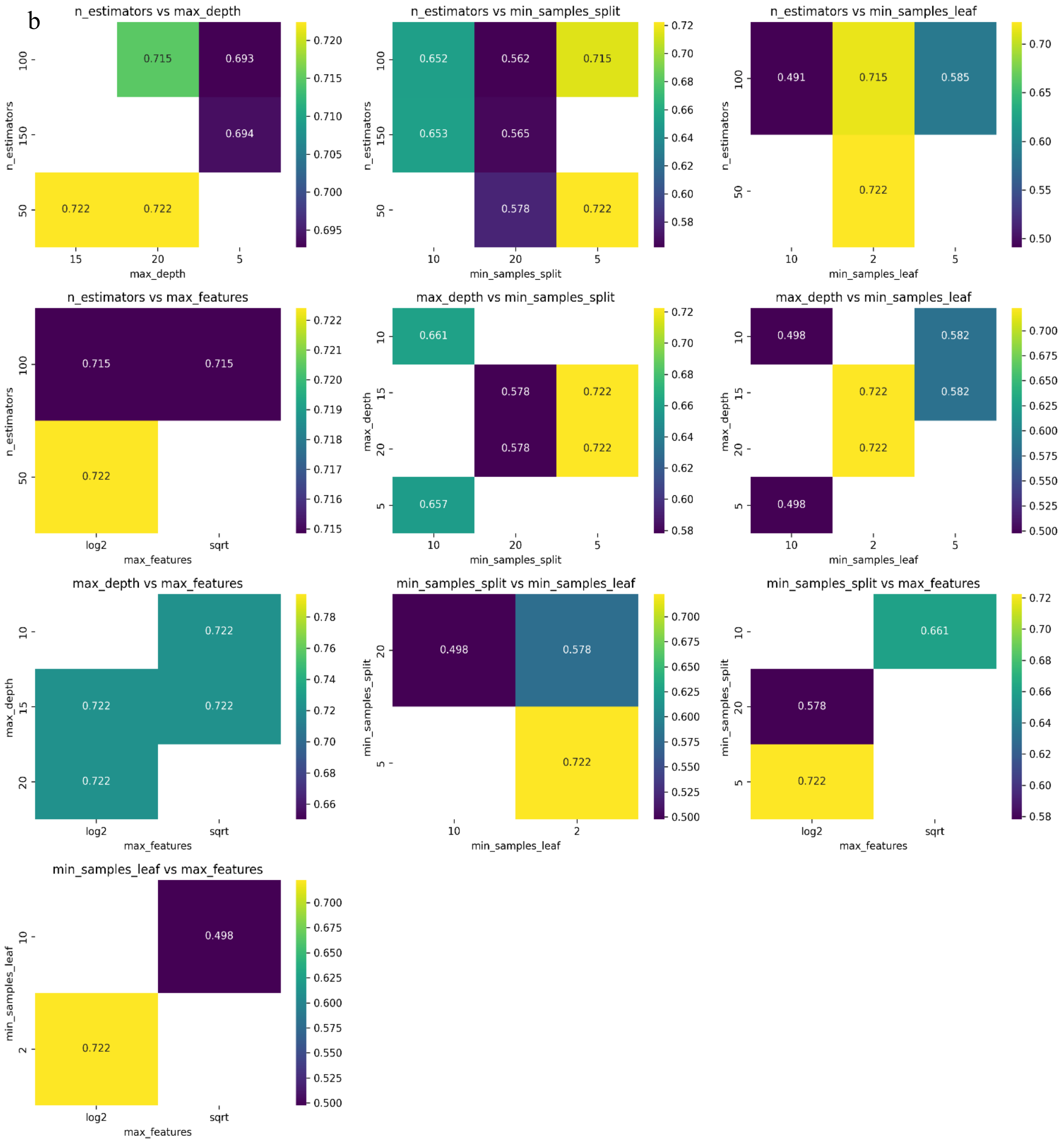
b
n_estimators vs max_depth
n_estimators
100
150
50
0.715
0.693
0.694
0.722
0.722
15
20
5
max_depth
0.720
0.715
0.710
0.705
0.700
0.695
n_estimators vs min_samples_split
n_estimators
0.652
0.562
0.715
0.653
0.565
0.578
0.722
10
20
5
min_samples_split
0.72
0.70
0.68
0.66
0.64
0.62
0.60
0.58
n_estimators vs min_samples_leaf
n_estimators
0.491
0.715
0.585
0.722
10
2
5
min_samples_leaf
0.70
0.65
0.60
0.55
0.50
n_estimators vs max_features
n_estimators
0.715
0.715
0.722
log2
sqrt
max_features
0.722
0.721
0.720
0.719
0.718
0.717
0.716
0.715
max_depth vs min_samples_split
max_depth
10
15
20
5
0.661
0.578
0.722
0.578
0.722
0.657
min_samples_split
max_depth vs min_samples_leaf
max_depth
0.498
0.582
0.722
0.582
0.722
0.498
min_samples_leaf
0.700
0.675
0.650
0.625
0.600
0.575
0.550
0.525
0.500
max_depth vs max_features
max_depth
0.722
0.722
0.722
0.722
max_features
0.78
0.76
0.74
0.72
0.70
0.68
0.66
min_samples_split vs min_samples_leaf
min_samples_split
0.498
0.578
0.722
min_samples_leaf
min_samples_split vs max_features
min_samples_split
0.661
0.578
0.722
max_features
min_samples_leaf vs max_features
min_samples_leaf
0.498
0.722
max_features

Explanations for Random Forest models

The hyperparameter optimization results for predicting the dimensionless area and maximum height of the fluidized zone using Random Forest models reveal consistent trends across both tasks. For A_f prediction, the highest R^2 score of 0.732 is achieved through configurations with moderately deep trees (max_depth = 10–15), a lower number of estimators (n_estimators = 50 or 150), and strict regularization (min_samples_split = 5, min_samples_leaf = 2). The use of "log2" for max_features appears universally beneficial, consistently yielding the best scores across different parameter combinations. These findings suggest that a well-regularized model with reduced feature sampling and shallow tree depth provides optimal generalization for predicting A_f.

Similarly, the H_f prediction task achieves a slightly lower but consistent peak score of 0.722, favoring the same pattern of hyperparameter values. Performance remains strong across a range of tree depths (10–20) and is particularly enhanced by the combination of min_samples_split = 5, min_samples_leaf = 2, and max_features = log2. Although both tasks benefit from similar configurations, the A_f model shows slightly higher stability and predictability. Overall, both heatmaps indicate that controlled tree growth, reduced feature randomness, and tight leaf and split constraints are key to maximizing Random Forest performance in modeling fluidized zone behavior.

Hyperparameter heat maps for Stacking model predicting the dimensionless fluidized area (A_f) a) SVR b) MLP

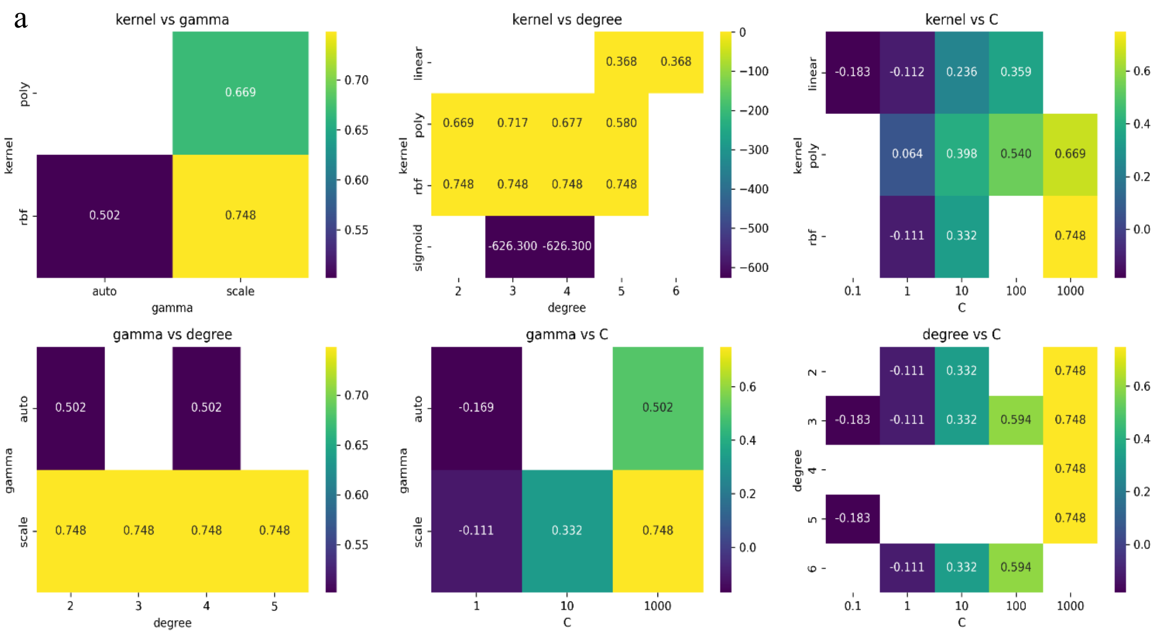

b

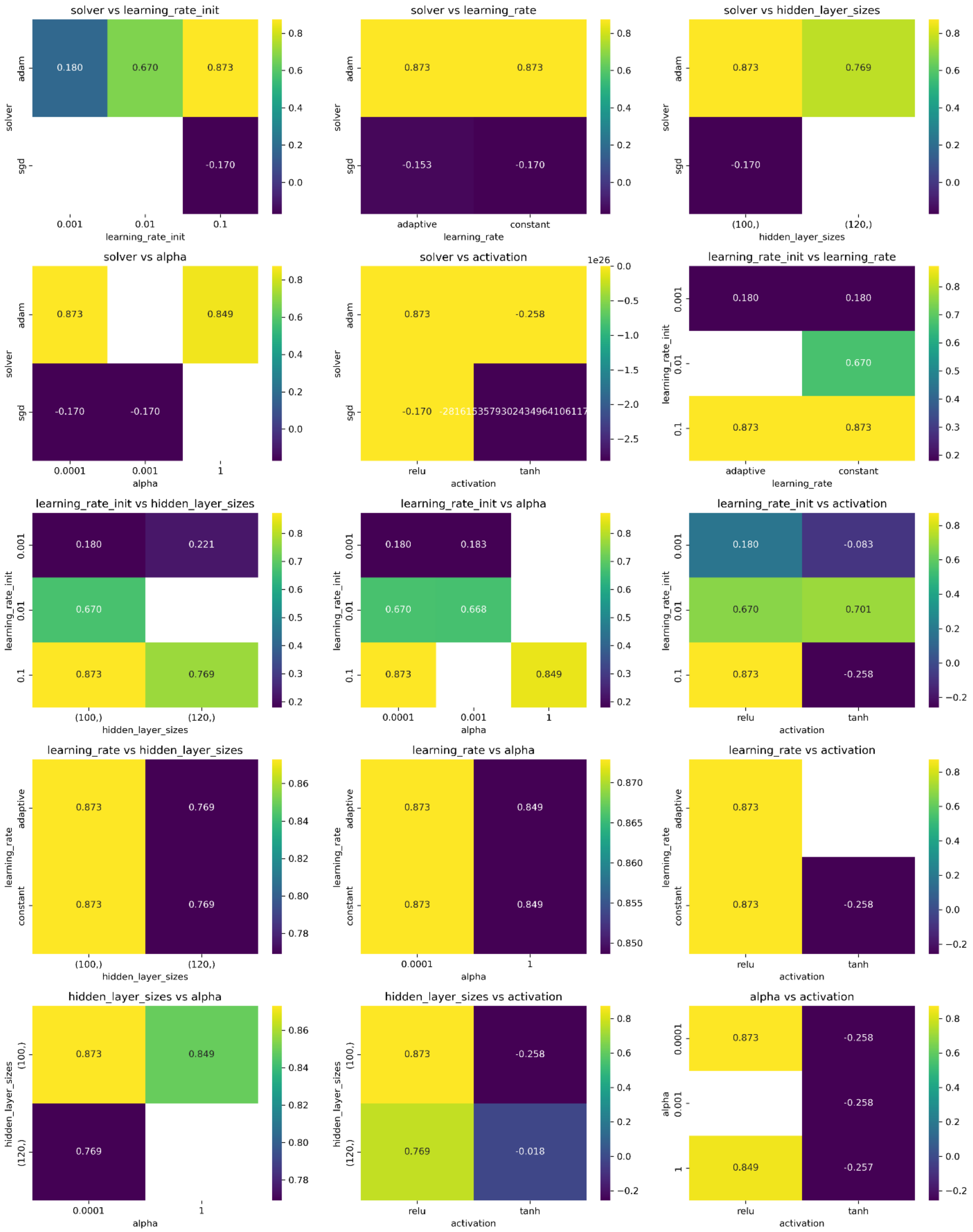
solver vs learning_rate_init
adam
sgd
solver
0.180
0.670
0.873
-0.170
0.001
0.01
0.1
learning_rate_init
solver vs learning_rate
0.873
0.873
-0.153
-0.170
adaptive
constant
learning_rate
solver vs hidden_layer_sizes
0.873
0.769
-0.170
(100,)
(120,)
hidden_layer_sizes
solver vs alpha
0.873
0.849
-0.170
-0.170
0.0001
0.001
1
alpha
solver vs activation
1e26
0.873
-0.258
-0.170
-2816153579302434964106117
relu
tanh
activation
learning_rate_init vs learning_rate
learning_rate_init
0.180
0.180
0.670
0.873
0.873
learning_rate_init vs hidden_layer_sizes
0.180
0.221
0.670
0.873
0.769
learning_rate_init vs alpha
0.180
0.183
0.670
0.668
0.873
0.849
learning_rate_init vs activation
0.180
-0.083
0.670
0.701
0.873
-0.258
learning_rate vs hidden_layer_sizes
learning_rate
0.873
0.769
0.873
0.769
learning_rate vs alpha
0.873
0.849
0.873
0.849
learning_rate vs activation
0.873
0.873
-0.258
hidden_layer_sizes vs alpha
hidden_layer_sizes
0.873
0.849
0.769
hidden_layer_sizes vs activation
0.873
-0.258
0.769
-0.018
alpha vs activation
0.873
-0.258
-0.258
0.849
-0.257

Explanation for Stacking model (MLP+SVR with linear regression metamodel) predicting dimensionless fluidized area (A_f)

The hyperparameter optimization heatmaps for the MLP model (Multilayer Perceptron) applied to the prediction of the dimensionless fluidized area (A_f) demonstrate that the most influential hyperparameters are solver, learning_rate_init, alpha, and the type of activation function. The highest performance ($R^2 \approx 0.873$) is achieved consistently when the adam solver is used in combination with a learning rate of 0.1, low regularization (alpha = 0.0001), and the Relu activation function. Furthermore, hidden layer sizes of (100,) outperform deeper configurations such as (120,). Poor scores are observed when using the sgd solver or tanh activation, indicating instability or underfitting. These patterns show that the MLP model benefits from adaptive, non-linear learning dynamics with shallow yet expressive architectures.

The SVR model (Support Vector Regression) heatmaps, which explore kernel types and regularization parameters for the same task, indicate that the optimal configuration uses the RBF kernel with a gamma setting of "scale" and a C value of 1000. This combination achieves the highest R^2 score of 0.748. In contrast, kernels like linear or sigmoid result in poor performance or even negative values, signaling overfitting or model failure. The results also show that polynomial kernels with degrees between 3 and 5 can perform moderately well, but they do not match the stability and predictive power of the RBF setup. When these two models (MLP and SVR) are stacked with a linear regression meta-model, they complement each other's strengths—MLP contributing deep nonlinear pattern learning and SVR offering robust margin-based fitting—resulting in a potentially more accurate and generalizable prediction framework for modeling fluidized zone characteristics.

Hyperparameter heat maps for Stacking model predicting the dimensionless fluidized height (H_f) a) SVR b) MLP

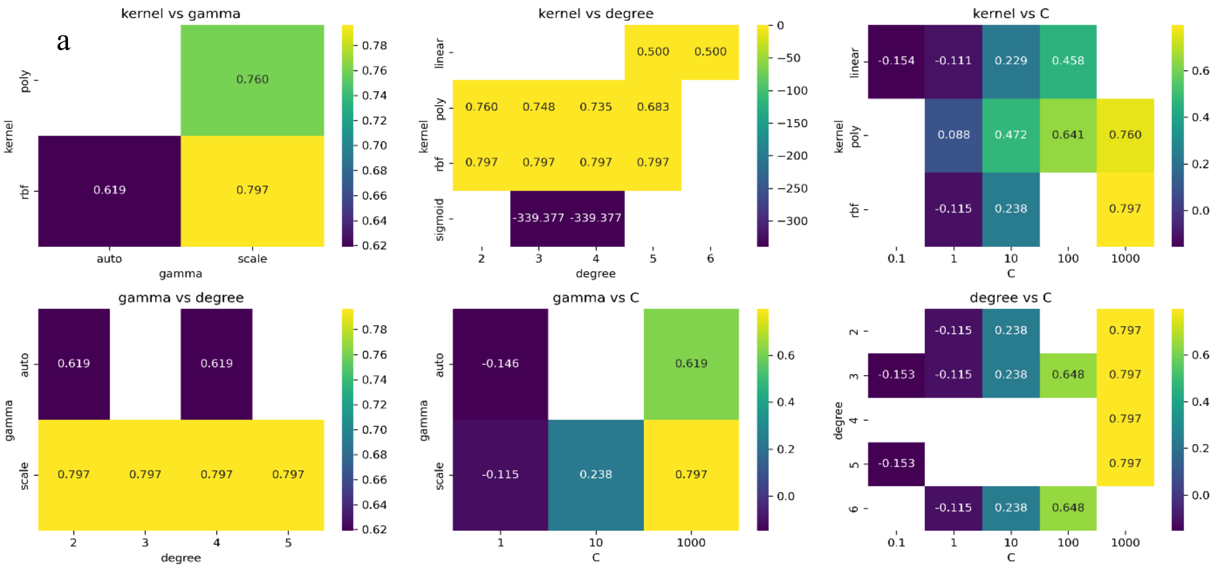

b

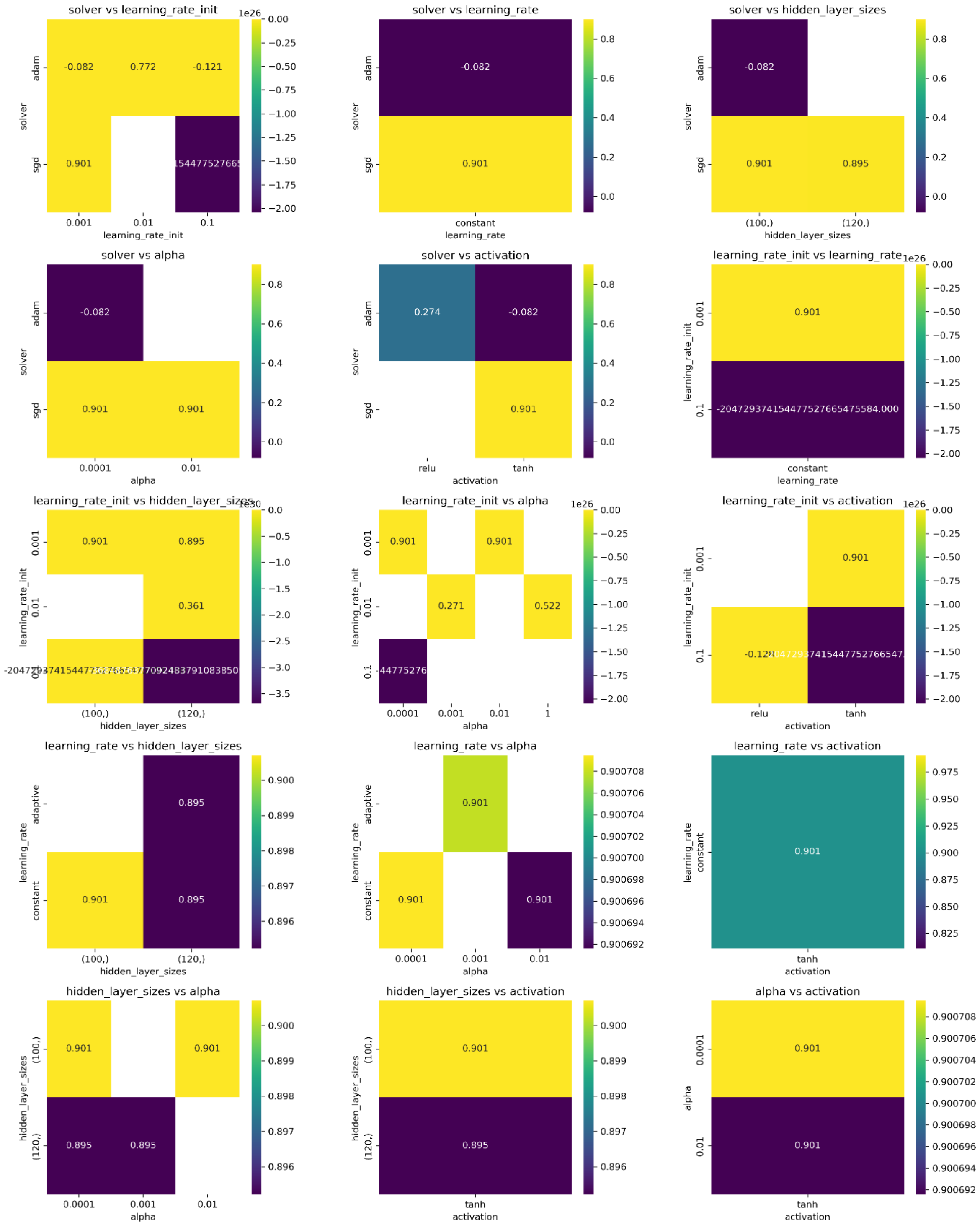
solver vs learning_rate_init
1e26
-0.082
0.772
-0.121
0.901
154477527665
solver
adam
sgd
0.001
0.01
0.1
learning_rate_init
solver vs learning_rate
-0.082
0.901
constant
learning_rate
solver vs hidden_layer_sizes
-0.082
0.901
0.895
(100,)
(120,)
hidden_layer_sizes
solver vs alpha
-0.082
0.901
0.901
0.0001
0.01
alpha
solver vs activation
0.274
-0.082
0.901
relu
tanh
activation
learning_rate_init vs learning_rate
1e26
0.901
-2047293741544775276654755584.000
learning_rate_init vs hidden_layer_sizes
1e30
0.901
0.895
0.361
learning_rate_init vs alpha
1e26
0.901
0.901
0.271
0.522
44775276
0.0001
0.001
0.01
1
learning_rate_init vs activation
1e26
0.901
learning_rate vs hidden_layer_sizes
adaptive
constant
0.895
0.901
0.895
learning_rate vs alpha
0.901
0.901
0.901
learning_rate vs activation
0.901
hidden_layer_sizes vs alpha
0.901
0.901
0.895
0.895
hidden_layer_sizes vs activation
0.901
0.895
alpha vs activation
0.901
0.901

Explanation for Stacking model (MLP+SVR with linear regression metamodel) predicting dimensionless fluidized height (H_f)

The hyperparameter optimization results for the MLP model applied to the prediction of dimensionless maximum fluidized height (Hf) show that the best performance ($R^2 \approx 0.901$) is consistently achieved when using the sgd solver in combination with a learning rate of 0.01, a constant learning schedule, and tanh as the activation function. The hidden layer sizes of (100,) also slightly outperform the larger structure (120,). Additionally, lower regularization values (alpha = 0.0001) tend to support better generalization. The heatmaps confirm that combinations involving the adam solver generally underperform in this context, highlighting the importance of simpler solvers and steady learning for capturing the dynamics associated with Hf. Unlike the Af prediction, Hf appears to benefit more from traditional training algorithms and consistent update rules.

For the SVR model targeting the same Hf variable, the results strongly favor the use of the RBF kernel with gamma set to "scale" and a large C value of 1000, which together yield the highest R^2 of 0.797. While the polynomial kernel also performs moderately well—especially for degrees between 3 and 5—the RBF configuration dominates in stability and score across multiple plots. The poor results associated with sigmoid and linear kernels (often yielding near-zero or even negative values) reinforce this preference. Additionally, the performance improves as the regularization parameter C increases, indicating that a softer margin with fewer constraints enhances the model's ability to capture the non-linear relationships involved in fluid height prediction. When MLP and SVR are combined into a stacking ensemble with a linear regression meta-model, the complementary learning styles—deep representation from MLP and margin-based generalization from SVR—are expected to synergize, leading to improved predictive performance for the Hf task.

Hyperparameter heatmap for RandomForest model predicting dimensionless a)A_f , b)H_f of fluidized zone

a

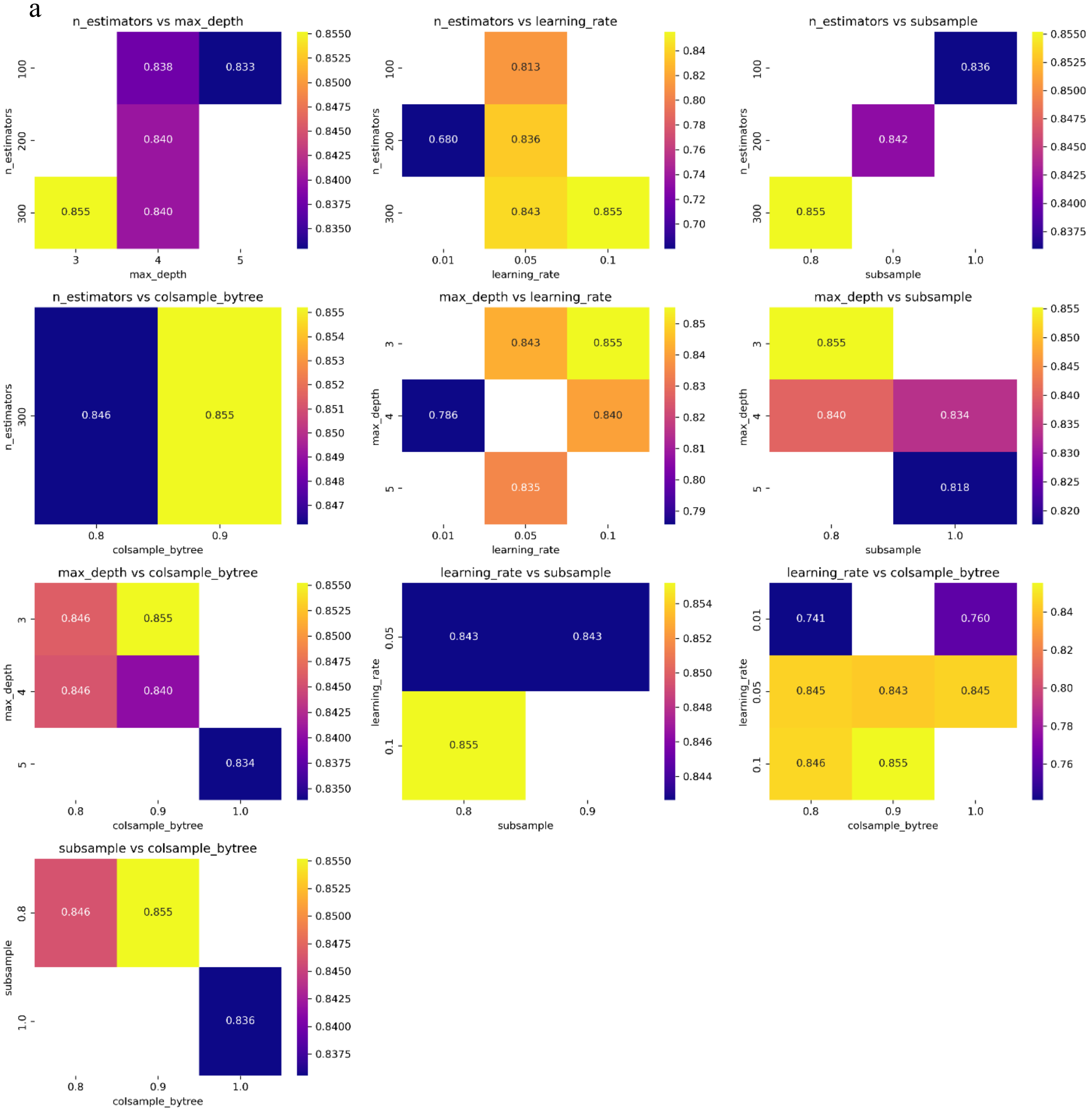

b

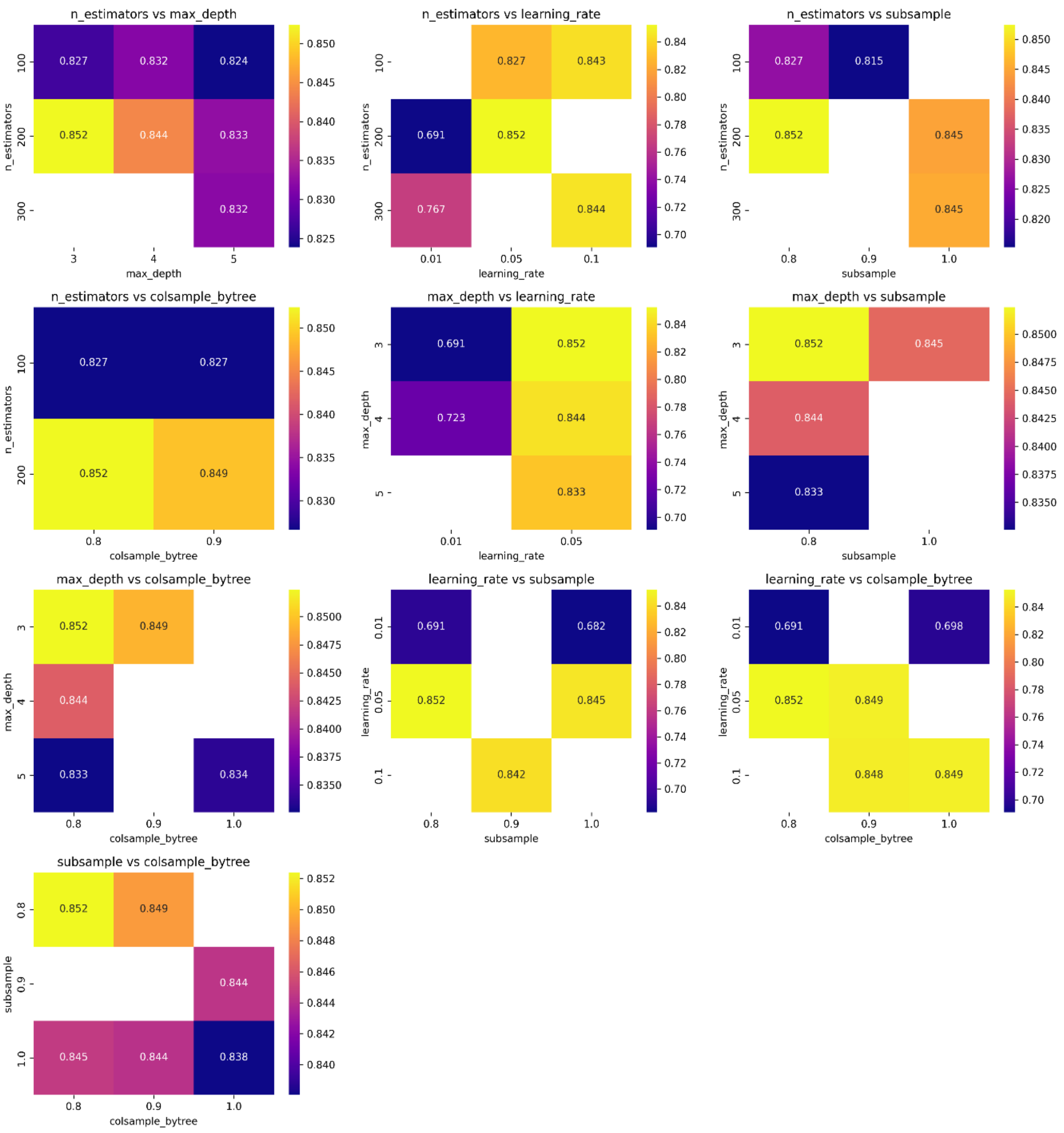

Explanations for XGBoost models

The hyperparameter optimization results for the XGBoost model used in predicting the dimensionless fluidized area show a clear pattern of high performance when specific parameter combinations are used. The best R^2 score observed is 0.855, and it is achieved across multiple subplots when the number of estimators is set to 300, the learning rate is 0.1, and the maximum depth is 3 or 4. Furthermore, the combinations involving subsample = 0.9 and colsample_bytree = 0.9 also consistently lead to optimal scores. These results highlight the importance of balanced tree depth and moderate

learning rates in capturing nonlinearities without overfitting. Also, the interaction between learning_rate and colsample_bytree confirms that slight regularization through column sampling (values around 0.9) improves generalization. Overall, the model is most effective when moderately deep trees (max_depth = 3 or 4) are combined with slower learning and partial subsampling strategies.

In the case of the XGBoost model for predicting the dimensionless maximum fluidized height (HF), a similarly strong pattern is observed with the highest R^2 value also reaching 0.852. This performance is obtained when n_estimators = 200, learning_rate = 0.1, max_depth = 3, and both subsample and colsample_bytree are in the range of 0.8 to 0.9. These heatmaps show that unlike in the AF case, adding more estimators slightly boosts performance for Hf prediction, possibly due to greater complexity in the height dynamics. Again, the learning rate of 0.1 proves optimal, reinforcing its general reliability for XGBoost on this domain. Notably, performance degrades at extreme subsample values (either too low or full sampling), suggesting that some randomness improves model robustness. In conclusion, both models benefit from similar tuning strategies, but the Hf model requires slightly more iterations to stabilize, while AF converges faster with simpler trees.
